# Plasmonic Nanoparticle-Enhanced Optical Techniques for Cancer Biomarker Sensing

**DOI:** 10.3390/bios13110977

**Published:** 2023-11-08

**Authors:** Li Fu, Cheng-Te Lin, Hassan Karimi-Maleh, Fei Chen, Shichao Zhao

**Affiliations:** 1 Key Laboratory of Novel Materials for Sensor of Zhejiang Province, College of Materials and Environmental Engineering, Hangzhou Dianzi University, Hangzhou 310018, China; feichen@hdu.edu.cn (F.C.); zhaoshichao@hdu.edu.cn (S.Z.); 2 Qianwan Institute, Ningbo Institute of Materials Technology and Engineering (NIMTE), Chinese Academy of Sciences, Ningbo 315201, China; linzhengde@nimte.ac.cn; 3 Key Laboratory of Marine Materials and Related Technologies, Zhejiang Key Laboratory of Marine Materials and Protective Technologies, Ningbo Institute of Materials Technology and Engineering (NIMTE), Chinese Academy of Sciences, Ningbo 315201, China; 4 University of Chinese Academy of Sciences, 19 A Yuquan Rd., Shijingshan District, Beijing 100049, China; 5 The Quzhou Affiliated Hospital of Wenzhou Medical University, Quzhou People’s Hospital, Wenzhou 325015, China; hassan@uestc.edu.cn; 6 School of Resources and Environment, University of Electronic Science and Technology of China, Chengdu 611731, China; 7 School of Engineering, Lebanese American University, Byblos 13-5053, Lebanon

**Keywords:** localized surface plasmon resonance, noble metal nanoparticles, cancer biomarkers, surface-enhanced Raman spectroscopy, photothermal/photoacoustic imaging, plasmonic metasurfaces

## Abstract

This review summarizes recent advances in leveraging localized surface plasmon resonance (LSPR) nanotechnology for sensitive cancer biomarker detection. LSPR arising from noble metal nanoparticles under light excitation enables the enhancement of various optical techniques, including surface-enhanced Raman spectroscopy (SERS), dark-field microscopy (DFM), photothermal imaging, and photoacoustic imaging. Nanoparticle engineering strategies are discussed to optimize LSPR for maximum signal amplification. SERS utilizes electromagnetic enhancement from plasmonic nanostructures to boost inherently weak Raman signals, enabling single-molecule sensitivity for detecting proteins, nucleic acids, and exosomes. DFM visualizes LSPR nanoparticles based on scattered light color, allowing for the ultrasensitive detection of cancer cells, microRNAs, and proteins. Photothermal imaging employs LSPR nanoparticles as contrast agents that convert light to heat, producing thermal images that highlight cancerous tissues. Photoacoustic imaging detects ultrasonic waves generated by LSPR nanoparticle photothermal expansion for deep-tissue imaging. The multiplexing capabilities of LSPR techniques and integration with microfluidics and point-of-care devices are reviewed. Remaining challenges, such as toxicity, standardization, and clinical sample analysis, are examined. Overall, LSPR nanotechnology shows tremendous potential for advancing cancer screening, diagnosis, and treatment monitoring through the integration of nanoparticle engineering, optical techniques, and microscale device platforms.

## 1. Introduction

Cancer remains one of the leading causes of death worldwide, accounting for nearly 10 million deaths in 2020 alone [1]. While survival rates have improved for certain cancer types with advances in treatment options, the early detection of cancer is still key for better prognosis and positive outcomes for patients [2]. The current clinical methods for cancer screening and diagnosis include imaging techniques such as magnetic resonance imaging (MRI), computed tomography (CT), and positron emission tomography (PET), as well as histopathology from tumor biopsies [3,4,5,6]. However, these conventional approaches lack adequate sensitivity and specificity, especially for detecting early-stage tumors and metastases. There is a critical need for new technologies that can detect cancer biomarkers with high precision and at ultra-low concentrations that are reflective of early disease.

The emerging field of nanotechnology offers exciting new possibilities for meeting this urgent need. Nanomaterials, with at least one dimension in the 1–100 nm range, have unique optical, electrical, and biological properties [7,8,9,10,11,12]. In particular, noble metal nanoparticles made from Au and Ag exhibit a phenomenon known as LSPR [13,14,15,16,17,18]. When the conduction electrons on the nanoparticle surface oscillate in resonance with visible or near-infrared light, they create strong electromagnetic fields that are confined or localized at the nanoparticle–dielectric interface [19,20,21,22]. The LSPR wavelength depends on factors such as nanoparticle composition, size, shape, and local dielectric environment [23,24]. By tuning these parameters, the optical absorption and scattering profiles of the nanoparticles can be precisely engineered. The light–matter interactions possible at the nanoscale through LSPR provide a powerful platform for developing ultrasensitive techniques to detect cancer biomarkers [25,26,27,28].

Some of the prominent LSPR-enhanced optical techniques that are revolutionizing cancer diagnostics include SERS [29], SPR [30], DFM [31], photothermal imaging [32], and photoacoustic imaging [33]. SERS utilizes electromagnetic enhancement from plasmonic nanostructures to boost the inherently weak Raman scattering signal of molecules, enabling single-molecule sensitivity. The molecular fingerprints provided by SERS allow for the multiplexed detection of protein, nucleic acid, and exosome biomarkers [34]. SPR detects changes in the refractive index near noble metal nanoparticle surfaces, which offers a label-free approach for detecting cancer biomarker binding events [35]. Combining plasmonic nanoparticles with SPR sensing leads to significant signal amplification. DFM visualizes LSPR nanoparticles based on the color of scattered light. Assemblies of nanoparticles scatter strongly, enabling the ultrasensitive detection of cancer cells, microRNAs (miRNA), and proteins [36]. Photothermal imaging employs LSPR nanoparticles as contrast agents that convert incident light to heat, producing thermal images that highlight cancerous tissues [37]. Photoacoustic imaging also involves the laser excitation of nanoparticles but detects ultrasonic waves generated by rapid thermoelastic expansion to achieve deeper tissue penetration [38]. Multiplexing capabilities are enabled by tuning the LSPR peaks of nanoparticles to different wavelengths [39]. Lab-on-a-chip devices and paper-based biosensors incorporate LSPR nanoparticles for point-of-care cancer detection [40].

In this review, we comprehensively survey the latest developments and future potential of LSPR nanotechnology for sensitive and specific cancer biomarker detection. We elucidate the fundamental LSPR phenomenon and principles of the key optical techniques being leveraged. We highlight innovative designs of plasmonic nanoparticles, substrates, and sensors, along with their applications. Remaining challenges that need to be addressed are examined, such as potential toxicity, lack of standardization, and practical issues with real clinical samples. We also discuss prospects for continued advances in LSPR nanotechnology and its integration into point-of-care diagnostics.

The insights provided in this review will be valuable across a diverse range of disciplines. For nanotechnology researchers, this will inspire new directions in nanoparticle synthesis and surface functionalization to create improved LSPR-based diagnostic agents. Biomedical engineers can gain perspectives on integrating LSPR techniques into practical lab-on-a-chip and portable devices. For clinicians and healthcare professionals, this will shed light on the tremendous potential of LSPR nanotechnology for advancing cancer screening, diagnosis, and treatment monitoring. Various key parameters are utilized to evaluate the performance of biosensors, including sensitivity, limit of detection, linear range, quality factor, enhancement factor (EF), reproducibility, and response time. To aid the reader’s understanding of this important terminology that will be referred to throughout the review, we provide concise definitions along with typical calculation/measurement methods for each parameter in Table 1.

Overall, this comprehensive overview of the field will help catalyze transdisciplinary efforts to accelerate clinical translation and make the long-envisioned promise of nanotechnology a widespread reality for game-changing cancer diagnostics.

## 2. Definition and Explanation of LSPR Phenomenon in Noble Metal Nanoparticles

LSPR is a phenomenon that arises in conductive nanoparticles when excited by light at specific wavelengths. While LSPR was first observed and described in noble metal nanoparticles such as gold and silver, further research has revealed that LSPR can also occur in nanoparticles made from other materials with suitable negative permittivity, including non-noble metals (e.g., aluminum, copper), doped semiconductors, and conductive metal oxides. However, noble metal nanoparticles remain the most commonly utilized for LSPR-based sensing due to their strong LSPR response in the visible and near-infrared regions, narrow LSPR bandwidth enabling high sensitivity, chemical stability allowing for surface functionalization, and synthetic tunability [41]. When nanoparticles made from gold, silver, or other suitable metals are irradiated with visible or near-infrared light matching their LSPR frequency, resonant oscillations of the conduction band electrons confined to the nanoparticle surface are induced [41]. The resonant oscillations arise from the collective oscillations of the free electrons in the metal nanoparticle. The geometry and dielectric environment of the nanoparticle determine the resonant conditions for the excitation of LSPR. These resonant plasma oscillations generate intense electromagnetic fields that extend outward from the nanoparticle surface into the surrounding medium, decaying evanescently away from the interface. Generally, the penetration depth of the evanescent field is on the order of tens to hundreds of nanometers, providing an intense localized electromagnetic field that can interact with and enhance the signature of target biomarker molecules within this distance [42].

**Figure 1:**
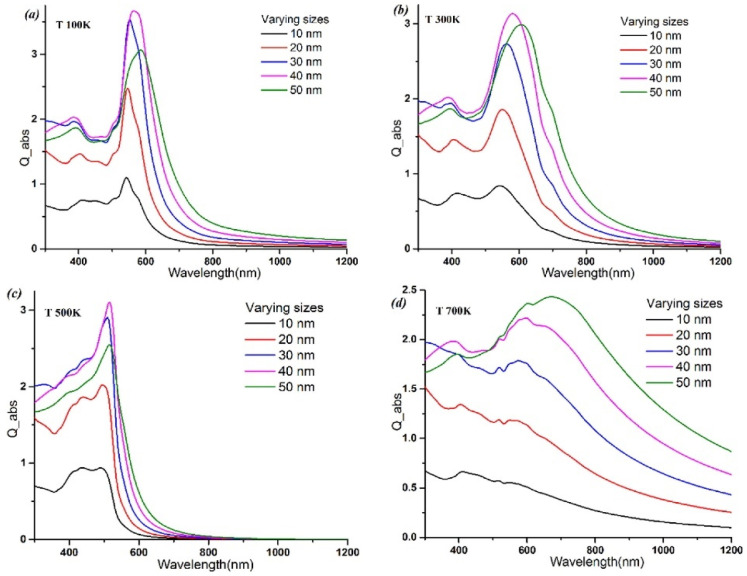
Absorption LSPR peak spectra as a function of wavelength of varying Au nanospheres at different temperatures in water embedded medium (*n* = 1.33) [44].

The resonant oscillation frequency, and hence LSPR wavelength, is dependent on the composition, size, shape, dielectric environment, and interparticle distance of the nanoparticles. By precisely engineering these parameters, the LSPR profile can be tuned to optimize nanoparticle interaction with light for boosting the inherently weak optical signals from biomarkers [43]. For example, the LSPR peak position of AuNPs red-shifts with increasing particle size for a given temperature (Figure 1). Bhatia and Verma [44] found that the absorption efficiency increases with particle size for all temperatures except for 50 nm nanoparticles at 700 K. They suggested that at higher temperatures, thermal oscillation can decrease the polarizability of nanoparticles, which would, in turn, decrease their absorption efficiency. Despite the decreased absorption efficiency at 700 K for 50 nm nanoparticles, their sensitivity as a refractive index sensor actually increases significantly at this temperature. The higher sensitivity is due to the larger shift in the LSPR peak position, with changes in the surrounding medium’s refractive index at elevated temperatures. Therefore, the 50 nm nanoparticles at 700 K, though exhibiting lower absorption, are actually more suitable for biosensing applications that require high refractive index sensitivity, while in the case of AgNPs, as the size of the AgNPs increases, there is a red-shift (shift to longer wavelengths) in the position of the LSPR peak. For example, as the size increased, the LSPR peak shifted from 390 nm to 460 nm. This demonstrates that larger AgNPs have LSPR peaks at longer wavelengths compared to smaller AgNPs [45]. The LSPR peaks of Au nanorods (AuNRs) can be widely tuned from the visible to near-infrared by changing the aspect ratio [46]. Au nanoshells, consisting of a dielectric core and ultrathin Au outer shell, have a highly red-shifted NIR LSPR that is determined mainly by the core/shell ratio [47].

The tunable LSPR offered by plasmonic noble metal nanoparticles makes them uniquely suited as contrast agents for cancer diagnostics based on various optical techniques [48]. Raman spectroscopy provides detailed molecular fingerprints based on inelastic light scattering, but its intrinsically low signal leads to poor sensitivity [49]. With SERS, the nanoparticles’ LSPR field enhances the Raman scattering to enable single-molecule detection [50]. For SPR and DFM, changes in the refractive index or light scattering around nanoparticles captured on sensor surfaces generate detectable optical signatures for binding events [51]. In photothermal and photoacoustic imaging, the nanoparticles’ efficient light-to-heat conversion or acoustic wave generation powered by LSPR gives high contrast [52].

Au and Ag are the predominantly used plasmonic materials for cancer diagnostics due to their stability and biocompatibility. Au is preferred for biomedical use because of the higher chemical stability. However, Ag exhibits a higher quality factor and stronger electromagnetic enhancement. Using bimetallic and core–shell nanoparticles that incorporate both Au and Ag is an effective approach to achieve both biocompatibility and optimal LSPR properties [53,54,55,56]. The core metal determines the dominant LSPR peak position, while the shell metal mainly enhances the intensity. Silica coating is often applied to noble metal nanoparticles to impart colloidal stability and allow surface bioconjugation while maintaining biocompatibility [57,58].

A key strategy to further enhance the sensitivity of LSPR techniques is to engineer assemblies of plasmonic nanoparticles with nanometer-scale gaps between particles. This enables electromagnetic “hot spots” where the LSPR fields couple and interact constructively, providing orders of magnitude signal enhancement over individual nanoparticles. For example, oligonucleotide or antibody linkers can attach AuNPs into assemblies with precise 2–10 nm gaps [59,60]. Lithographic fabrication methods can create arrays of nanoparticle pairs with tunable spacing on sensor chips [61,62]. DNA origami and molecular templating approaches guide the self-assembly of complex multi-nanoparticle clusters [63,64,65]. The coupled LSPR leads to SERS EF on the order of 10^8^–10^10^ in these hot spots.

Another strategy is to fabricate hybrid nanoparticle structures combining noble metals with other functional materials. AuNPs or AgNPs adsorbed onto graphene oxide surfaces exhibit stronger SERS due to interactions between their LSPR fields [66,67]. Semi-conductor quantum dots coupled to Ag nanoparticles also show enhanced photoluminescence and SERS via plasmon–exciton interactions [68]. Local heating in AuNPs attached to thermo-responsive polymer capsules has been leveraged for controlled drug release [69,70]. These examples illustrate the broad potential of hybrid LSPR nanosystems for theranostics—simultaneous diagnostics and therapy.

**Figure 2:**
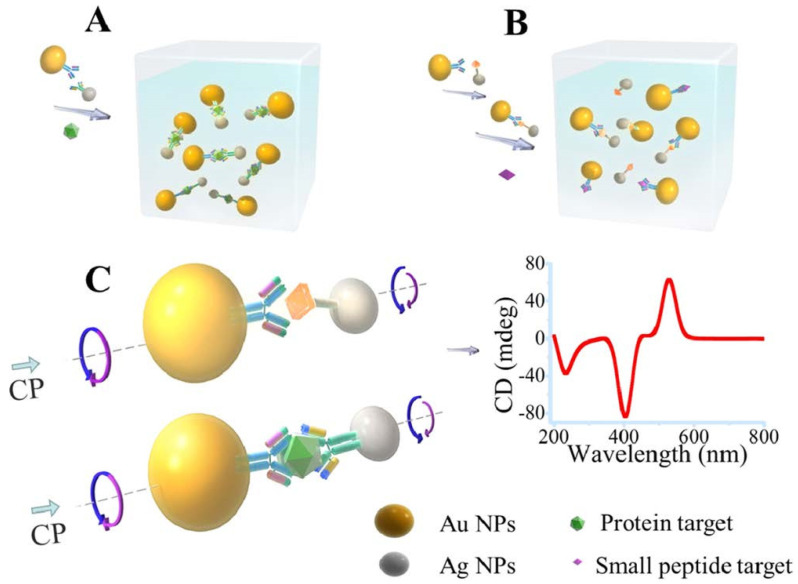
(**A**) The nanoparticle dimer was assembled from Au NPs and Ag NPs, which were functionalized with complementary biomacromolecules. (**B**) For the detection of small peptides, exemplified by microcystin-LR, the competitive immunorecognition assay was chosen to demonstrate its applicability to biological analysis. It results in a decrease of the CD amplitude. (**C**) For detection of the fairly large proteins exemplified by PSA, we used sandwich immunoassay mode. Schematics of the nanoparticle dimers bridged by immunocomplexes were used in competitive and sandwich immunoassays [79].

A key emerging direction is the use of optically active metasurfaces constructed using LSPR nanoparticles instead of continuous metallic films. Metasurfaces with nanoparticle arrays exhibit resonance properties strongly dependent on lattice parameters and geometry. This enables greater control over actively tunable plasmonic effects, such as Fano resonances and optical chirality [71,72,73,74,75,76]. Chiral objects derive their optical activity from their asymmetric 3D structure. When light interacts with a chiral object, the left and right circularly polarized components of the light interact differently, resulting in the rotation of the plane of polarization. However, some non-chiral objects can also be optically active if they lack mirror symmetry in three dimensions. New sensing modalities can also be unlocked. For example, chiral plasmonic metasurfaces enable detecting cancer biomarker binding by monitoring changes in optical chirality using circular dichroism spectroscopy [77,78]. Wu et al. [79] demonstrated that pairs of AuNPs and AgNPs connected by biological bridges exhibit unexpected chirality, enabling ultrasensitive chiroplasmonic bioanalysis of prostate-specific antigen (PSA). They formed dimers consisting of one antibody-functionalized AuNP and one competitive antigen-modified AgNP. Surprisingly, these simple nanoparticle pairs showed intense circular dichroism signals, indicating chirality (Figure 2). This was unexpected since spherical nanoparticles cannot be chiral (the combination of spherical nanoparticles with chiral molecules might lead to the enhancement of the molecular CD signal [78]). Further analyses revealed that the chirality originated from the elongated, non-spherical shape of the individual nanoparticles. When assembled into dimers, the long nanoparticle axes aligned at a consistent 9° angle, forming a twisted, scissor-like geometry that breaks symmetry. Moreover, the sign of the chirality was consistent due to the specific conformation of the biological bridges. This simple yet robust nanoparticle system enabled sensitive detection of a toxin peptide and cancer biomarker at record low detection limits. They achieved one to two orders of magnitude improvement over other techniques by exploiting three chiroplasmonic effects: (1) the plasmonic enhancement of intrinsic biomolecule chirality, (2) strong optical coupling from the twisted nanoparticle geometry, and (3) signal amplification from the bisignate CD bands.

**Figure 3:**
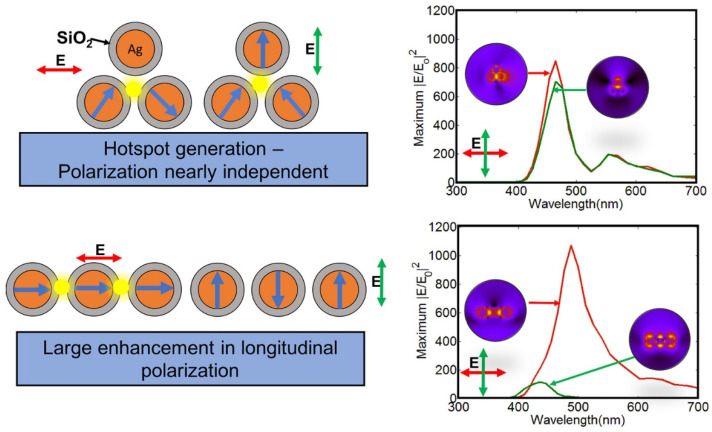
Schematic of the Ag@SiO_2_ core–shell trimers with different geometries modeled using FDTD simulations [80].

Theoretical modeling using finite-difference time-domain (FDTD) and finite element methods has been invaluable to guide the design of LSPR nanoparticles and assemblies. The simulation of the interactions between light and nanostructures provides insights into photonic confinement effects and near-field enhancement that are difficult to probe experimentally. This facilitates the rational optimization of factors such as nanoparticle shape, assembly geometry, spacer material, and linker orientation to strategically harness electromagnetic hot spots for maximizing detection sensitivity. For example, Nagarajan et al. [80] used 3D FDTD simulations to analyze the plasmonic properties and electric field enhancement of Ag@SiO_2_ core–shell trimers (Figure 3). Specifically, the authors looked at how the geometry and symmetry breaking of the core–shell trimers affected LSPR wavelength and electric field enhancement. They modeled trimers with different vertex angles, from an equilateral triangle (60°) to a linear chain (180°). The FDTD simulations showed that the highly symmetric equilateral triangle trimers exhibited degenerate or identical LSPR wavelengths for the two orthogonal polarizations studied. They also showed intense electric field hotspots in the junction region between the nanospheres. As the symmetry was broken by increasing the vertex angle, this degeneracy was lifted, and the LSPR wavelengths were split for the two polarizations. The electric field enhancement also changed, with the linear chain trimers demonstrating the highest enhancement, around four times greater than bare metal trimers. However, the linear chain structures were also much more sensitive to polarization direction. The LSPR red-shifted and blue-shifted for longitudinal and transverse polarization, respectively. In contrast, the symmetric equilateral trimers showed large, polarization-insensitive field enhancement. The authors explained that the LSPR shifts and electric field changes upon symmetry breaking using plasmon hybridization theory. This theory models the plasmon modes of complex nanostructures like the trimers as hybrids of the plasmon modes of individual nanoparticles.

LSPR is a versatile phenomenon unique to plasmonic nanostructures that can be leveraged for enabling and enhancing diverse optical techniques to push the frontiers of cancer biomarker detection. The combination of synthetic nanoparticle engineering and computational nano-photonics modeling has fueled tremendous advances in constructing ultrasensitive LSPR-based detection platforms. Ongoing research is focused on investigating new nanoparticle compositions, such as alternative plasmonic metals and alloys, unconventional geometries using top-down and bottom-up assembly, and hybrid metasurfaces with tailored resonances. These efforts promise to open up even greater opportunities and capabilities in LSPR nanotechnology for early cancer diagnosis, treatment monitoring, and precision medicine.

## 3. Surface-Enhanced Raman Spectroscopy (SERS)

### 3.1. Concepts of SERS

SERS has emerged as a powerful technique for the sensitive and multiplexed detection of cancer biomarkers by harnessing the strong electromagnetic field enhancement provided by the LSPR of noble metal nanostructures. SERS overcomes the main limitation of conventional Raman spectroscopy, which is the extremely weak Raman scattering signal, to enable detection down to single-molecule sensitivity. The rich molecular vibrational information offered by Raman spectra allows for the highly specific identification of target biomarkers. Moreover, SERS offers key advantages, including narrow spectral bands, multiplexing capabilities, and no photobleaching issues compared to fluorescence techniques [81].

The EF provided by SERS substrates can be as high as 10^8^–10^11^ using rationally designed plasmonic nanostructures, allowing for the detection of biomolecules at ultra-low concentrations [82]. There are two mechanisms responsible for the enhancement—electromagnetic (EM) enhancement and chemical (CM) enhancement [83]. EM enhancement is mediated by the strong localized electric fields generated at the nanoparticle surface upon excitation of LSPR. CM enhancement arises from charge transfer between the nanoparticle and adsorbed molecule. Though weaker, CM enhances the polarizability of the molecule to further boost the Raman signal [84]. The EM enhancement mechanism contributes the majority of the overall SERS EF. Optimized nanostructures can maximize this EM enhancement, which acts as the primary “amplification” that allows SERS to detect extremely low concentrations of molecules. The CM enhancement mechanism provides a smaller but still significant contribution to the overall EF through charge transfer and interactions between molecules and nanoparticles.

### 3.2. Direct and Indirect Detection of Cancer Biomarkers Using SERS

**Figure 4:**
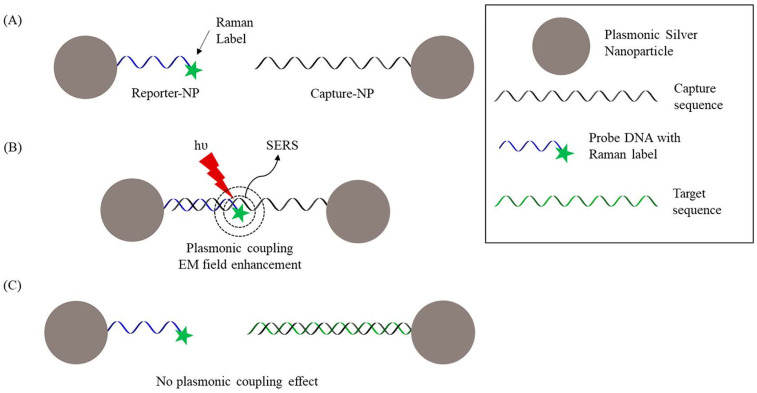
Schematic showing functionalized silver NPs and the detection mechanism of the PCI strategy. (**A**) Capture-NPs and Raman-labeled reporter-NPs are synthesized by functionalizing AgNPs with thiolated complementary DNA probes. (**B**) Plasmonic coupling is induced in a three-dimensional nanonetwork cross-linked by capture/reporter DNA duplexes that have the Raman label located between adjacent nanoparticles. (**C**) In the presence of target strands, targets compete with reporter-NPs for binding to capture-NPs, thereby preventing the formation of nanonetworks, leading to a significantly reduced plasmonic coupling effect [85].

There are two main approaches to detecting cancer biomarkers using SERS—direct and indirect detection. Direct detection involves analyzing the intrinsic SERS spectral signatures of biomarker proteins adsorbed onto plasmonic nanoparticles or nanostructured surfaces [85]. For example, Wang et al. [85] developed a SERS-based approach called plasmonic coupling interference (PCI) to detect cancer-related miRNA biomarkers without the need for extrinsic labels. The PCI method works by assembling silver nanoparticles functionalized with DNA probes into three-dimensional networks, with Raman reporter molecules incorporated between adjacent nanoparticles. When the nanoparticles are in close proximity, plasmonic coupling greatly amplifies the Raman signals of the reporter molecules. The addition of target miRNA biomolecules inhibits network formation by binding to the DNA probes, thereby reducing Raman amplification (Figure 4). The researchers demonstrated multiplex detection of four important miRNA biomarkers linked to cancer—miR-149, miR-125b, let-7a, and miR-21. For each target, they designed complementary DNA probes to assemble nanoparticle networks. In the presence of target miRNA, the SERS signal decreased in a concentration-dependent manner with detection limits in the low nanomolar range. Control experiments with non-complementary miRNAs showed excellent specificity. Further theoretical analysis revealed that nanoparticle dimerization with a Raman label in between can enhance Raman signals over 1000-fold compared to isolated nanoparticles. This label-free approach provides detailed molecular structural information but is only applicable to certain proteins with high affinities for the SERS substrate. For example, Lu et al. [86] developed a label-free SERS method for the sensitive detection of nucleoside diphosphate kinase. He et al. [87] recently reported a detection of kinase A suing a label-free SERS platform. It also requires extensive purification, which limits practicality.

**Figure 5:**
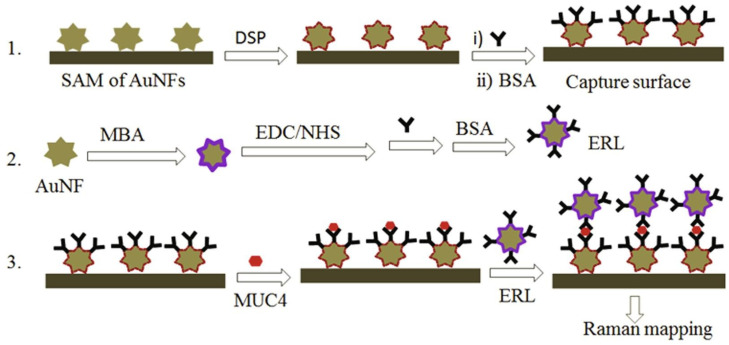
Scheme showing the sandwich immunoassay procedures (MBA: 4-mercaptobenzoic acid; BSA: bovine serum albumin; DSP: dithiobis (succinimidyl propionate); ERL: extrinsic Raman label; EDC/NHS: cross-linkers) [88].

The indirect approach uses SERS nanotags labeled with Raman reporter dyes and functionalized with recognition elements such as antibodies to specifically bind biomarkers. For example, Bedemo et al. [88] developed a highly sensitive and reliable Au nanoflower (AuNF)-based SERS detection method for the pancreatic cancer biomarker MUC4. The authors used an indirect sandwich immunoassay approach, where the analyte MUC4 is captured on a surface and then detected using a SERS-active nanotag label (Figure 5). Specifically, they designed a uniform SERS-active substrate by immobilizing AuNFs on a thiol-functionalized silicon wafer to serve as the capture surface. The AuNFs were optimized for maximum SERS enhancement. The capture antibodies were then immobilized to capture MUC4. For detection, they designed a SERS-active nanotag extrinsic Raman label (ERL) by conjugating Raman reporter molecules and detection antibodies onto AuNFs. In the assay, different concentrations of MUC4 samples were incubated on the capture surface, allowing the MUC4 to be captured by the surface antibodies. The ERLs were then added, which bind to the captured MUC4. The key finding was that this approach could detect MUC4 at an ultralow concentration down to 0.1 ng/mL. The indirect detection of MUC4 using the ERL, rather than directly detecting the MUC4 itself, was critical for achieving high sensitivity. The SERS enhancement provided by the AuNF nanotags greatly boosted the Raman signal of the reporter compared to directly detecting the inherently weak Raman signal of MUC4. Using AuNFs for both the capture substrate and ERL enabled strong SERS enhancement in both stages. In another example, Verdin et al. [89] demonstrated a multiplex micro-SERS imaging technique using functionalized Au–Ag core–shell nanoparticles to evaluate the expression of two important cancer biomarkers—folate receptors (FR) and sialic acid (SA)—in both cancer cells and tissues. The authors developed two types of SERS nanoprobes, each targeting either FR or SA, by coating the nanoparticles with Raman reporter molecules and a polymer layer of poly(allylamine). The polymer layer served to stabilize the Raman signal and enable functionalization with small targeting molecules—folic acid for FR and 4-carboxyphenylboronic acid for SA. These inexpensive targeting molecules provided specificity toward the cancer biomarkers while maintaining stability. The FR- and SA-targeting nanoprobes showed strong SERS signals and specificity toward cancer cell lines overexpressing FR or SA compared to control cells. Micro-SERS imaging of breast and ovarian cancer tissues revealed a higher expression of both biomarkers in tumor regions versus healthy regions. The multiplex imaging technique allowed for the simultaneous evaluation of FR and SA expression patterns in the same tissue section. Quantification of the SERS signal revealed up to a four-fold higher expression of FR and SA in tumor areas compared to healthy areas. For cellular and tissue experiments, multiple samples were analyzed using the SERS probes. The authors show consistent trends in SERS signal differences between cancerous and non-cancerous cells/tissues across these replicates. This indirect approach of using SERS nanoprobes and micro-SERS imaging enabled sensitive and multiplex visualization of cancer biomarker expression in clinically relevant samples with high spatial resolution.

### 3.3. Materials Used for SERS

Au and Ag are the predominantly used plasmonic materials for SERS, with Au being favored for biomedical applications due to higher chemical stability. SERS-active nanoparticles utilized include nanospheres [90], NRs [91], nanostars (NSs) [92], nanoplates [93], and core–shell structures [94], whose LSPR peaks can be tuned based on the shape. Ag and Au nanocubes are also excellent SERS substrates due to the EM enhancement at sharp corners. SERS enhancement can be further optimized using nanoparticle assemblies and nanoaggregates that enable intense hot spots in the nanogaps between particles. Arrays of nanopillars [95], encapsulated shell–core–shell nanoparticles [96], and origami nanostructures [97] create internal hot spots within nanostructures for reproducible EF.

For multiplexed detection, mixed assemblies of nanoparticles labeled with different Raman reporters, such as fluorescent dyes, or batch synthesis of nanoparticles encapsulating diverse reporters, allows for a barcode-type SERS spectral signature [98,99,100]. Common reporters include non-thiolated aromatic dyes, such as rhodamine [101], crystal violet [102], and malachine green [103], which adsorb onto nanoparticles via hydrophobic and electrostatic interactions. Thiolated aromatics such as mercaptobenzoic acid derivates that bind covalently are also popular choices [100]. Quantum dots [98], carbon nanotubes [104], graphene [105], and other nanocarbons [106] are alternative SERS reporters under investigation.

**Figure 6:**
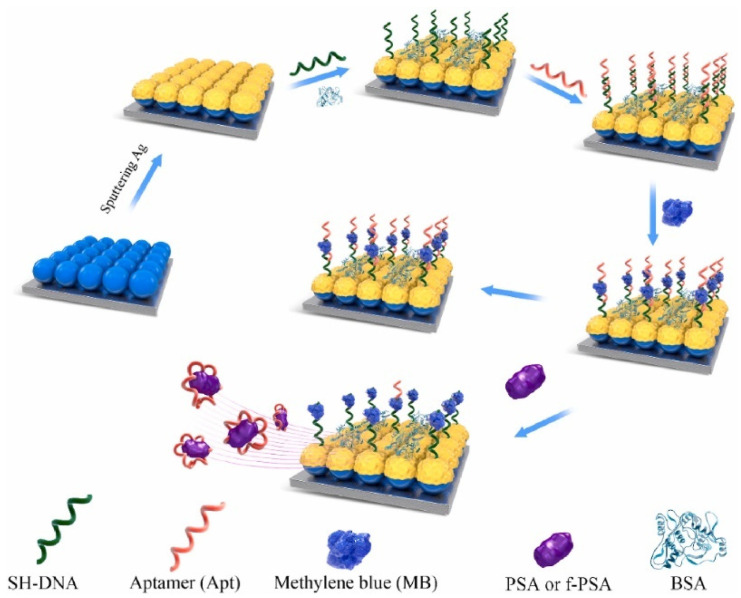
Fabrication procedure of the SERS-based aptasensor for the detection of PSA and f-PSA [108].

SERS-based immunoassays employ a sandwich format using plasmonic nanoparticles functionalized with Raman reporters and an antibody that binds the target protein biomarker. Capture antibodies immobilized on a surface then bind the protein target, followed by SERS nanoparticle binding for signal enhancement. This allows for the multiplexed detection of panels of protein biomarkers using different SERS nanotags. For example, Banaei et al. [107] developed a SERS-based sandwich immunoassay platform in a microfluidic chip to quantify multiple protein biomarkers associated with pancreatic cancer, ovarian cancer, and pancreatitis (CA19-9, HE4, MUC4, MMP7, and mesothelin). The purpose of using a sandwich immunoassay format was to amplify the SERS signal and enable the sensitive, reproducible detection of the biomarkers. Au nanoshells functionalized with Raman reporter molecules and detection antibodies were used to generate SERS signals specific to each biomarker captured on the chip surface. The microfluidic platform improved reproducibility compared to conventional SERS assays. The expression levels of all five biomarkers were measured in patient serum samples from the three disease groups and healthy controls. Then, machine learning algorithms, including K-nearest neighbor classification and decision tree analysis, were applied to the multiple biomarker datasets. This allowed for discrimination between diseases with overlapping single biomarker changes. The machine learning models improved specificity significantly compared to relying on individual biomarkers for diagnosis. In particular, the combination of the microfluidic SERS assay and machine learning could differentiate between pancreatic cancer, ovarian cancer, and pancreatitis. This demonstrates a convenient liquid biopsy approach with high specificity for cancer screening and early diagnosis. The key innovations lie in the integration of multiplex SERS microfluidics with machine learning, which together enable rapid and highly specific discrimination between cancers with common biomarker profiles. Such assays have reached sensitivities down to femtomolar levels, rivaling established clinical assays such as ELISA. SERS nanotags linked to aptamers or affibodies instead of antibodies have also been applied, taking advantage of their stability and multiple binding orientations. However, the researchers also acknowledge some limitations in the repeatability of their sensor. The coefficient of variation (CV) of the Raman intensity measurements was still around 20–30%, even with the microfluidic chip, indicating that there is still room for improvement in repeatability. The small size of their dataset (only 20 serum samples in total) limited their ability to more accurately estimate the test error of their machine learning models. For example, Zhao et al. [108] reported a highly sensitive and selective biosensor for detecting prostate cancer biomarkers in serum samples. The authors fabricated a SERS-based aptasensor using polystyrene–AgNP arrays as a uniform SERS-active substrate. The sensor detects the total PSA and free PSA (f-PSA), which are overexpressed in prostate cancer patients. The aptasensor was constructed by immobilizing a PSA-specific aptamer and its complementary DNA probe on the SERS substrate. The Raman reporter methylene blue was then bound to the aptamer (Figure 6). In the presence of PSA, the target binds to the aptamer, releasing the methylene blue into the solution and decreasing the SERS signal. This allows PSA levels to be quantified through the SERS signal change. The RSD of the peak intensity at 1623 cm^−1^ when measuring 1 ng/mL PSA was reported to be 4.41% for 10 random Raman spectra collected from the substrate. The sensor demonstrated high reproducibility, selectivity for PSA over other proteins, and sensitivity down to 0.01 ng/mL for PSA and 0.25 ng/mL for f-PSA. Testing in clinical serum samples from prostate cancer patients confirmed the sensor’s ability to accurately detect PSA biomarkers at clinically relevant concentrations.

For detecting nucleic acid biomarkers, such as circulating tumor DNA (ctDNA) and miRNAs, SERS nanotags functionalized with complementary DNA or RNA probes have been developed. The strong Raman enhancement enables detection at a single-base mutation sensitivity. For example, Zhang et al. [109] demonstrated a highly sensitive method for detecting ctDNA in lung cancer patients using SERS. The authors developed a SERS-based assay using a hairpin DNA–RNA–DNA probe to specifically recognize the target ctDNA biomarker. The key innovation enabling this ultrasensitive detection is the use of the RNase HII enzyme to amplify the signal. The hairpin probe contains an RNA segment that is selectively cleaved when hybridized to the target ctDNA. This cleavage releases the ctDNA to bind new probes, cycling the target and amplifying the signal. For SERS detection, the Raman reporter 5,5′-dithiobis(succinimidyl-2-nitrobenzoate) was attached to the DNA probe. After RNase HII cleaves the RNA site, the remaining DNA fragment binds to a complementary DNA sequence, causing a red-shift in DSNB’s nitro vibrations. By measuring this SERS frequency shift, they could detect ctDNA down to femtomolar levels. The assay achieved a limit of detection of 1.2 × 10^−16^ M for a mutant KRAS ctDNA sequence, a clinically relevant biomarker in lung cancer. This is a 100–1000 fold improvement over traditional SERS sandwich assays. The SERS sensor showed good repeatability and reproducibility, with RSDs of <3.6% for Raman intensity and standard deviations of ~0.04 cm^−1^ for frequency shifts.

**Figure 7:**
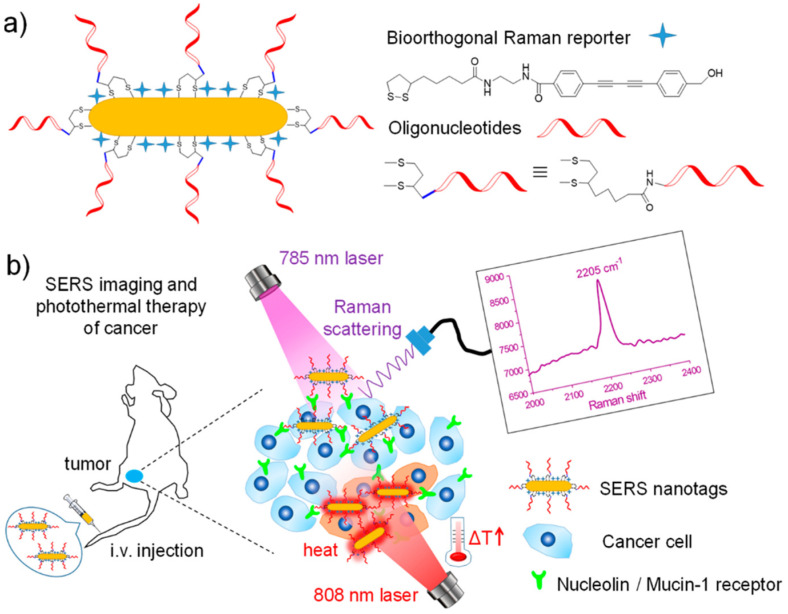
(**a**) Fabrication of oligonucleotide-modified bioorthogonal SERS nanotags. (**b**) Bioorthogonal SERS nanotags as a precision theranostic platform for cancer detection and photothermal therapy in mice after intravenous injection [110].

Photothermal SERS nanoparticles that heat up upon laser irradiation have been created for simultaneous biomarker detection and hyperthermal tumor ablation. A novel bioorthogonal SERS nanotag has been fabricated for both highly specific cancer detection and photothermal therapy [110]. The authors functionalized AuNRs with a custom Raman reporter containing a diynl group and aptamers that target cancer biomarkers. When irradiated with a near-infrared laser, the AuNRs generate heat that can be used to kill cancer cells. At the same time, the diynl group on the Raman reporter generates a strong SERS signal at 2205 cm^−1^, a biologically silent region, allowing for the highly sensitive and specific detection of the cancer biomarkers (Figure 7). In cell studies, the aptamer-functionalized SERS nanotags specifically targeted cancer cells overexpressing the biomarkers. Irradiating the cells with a laser induced significant toxicity due to the photothermal effect of the AuNRs. In mouse models, the nanotags allowed for the highly specific SERS detection of tumors after intravenous injection. With laser irradiation, the nanotags achieved 99% tumor growth inhibition and no observable toxicity.

### 3.4. SERS Imaging

The imaging capabilities of SERS make it a powerful tool for cancer diagnostics and intraoperative guidance. The spatial mapping of tumor margins in excised tissues using topically applied SERS nanoparticles that target overexpressed proteins has been demonstrated. Li et al. [111] demonstrated a SERS platform for the targeted imaging of prostate cancer cells. The authors developed optimized AuNS-based SERS tags coated with 4-nitrothiophenol and a protective silica layer. These SERS tags were then functionalized with a small molecule inhibitor of prostate-specific membrane antigen (PSMA) to enable specific targeting to prostate cancer cells. PSMA was chosen as the target because it is overexpressed in advanced, metastatic prostate cancers. The authors showed that by carefully designing the plasmonic properties of the AuNSs, they could create SERS tags with intense Raman signals and ultra-high binding affinity to PSMA—around four orders of magnitude higher than existing clinical imaging agents. Using these optimized SERS tags conjugated to the PSMA inhibitor, the authors were able to selectively detect and image PSMA-expressing prostate cancer cells with very high sensitivity. They demonstrated that their approach enabled quantitative, stable Raman spectral measurements of the targeted cells using Raman microscopy. By comparing prostate cancer cell lines with and without PSMA expression, they showed that the SERS agent could discriminate between phenotypes with high specificity. Remarkably, concentrations as low as 20 pM of the targeted SERS agent were sufficient to generate a detectable signal for site-selective recognition of prostate cancer cells.

**Figure 8:**
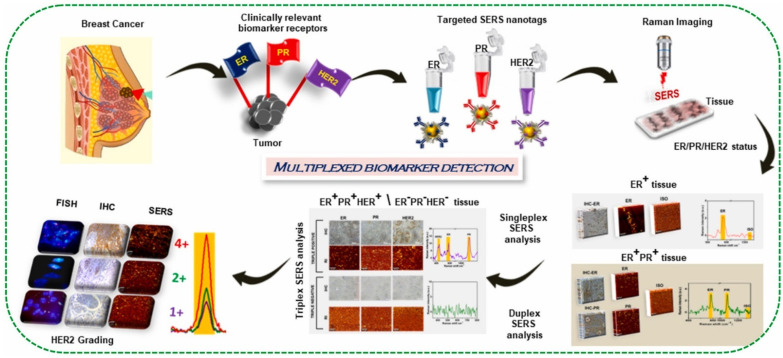
Raman spectroscopy-driven histopathologic approach was evolved for clinical diagnostics utilizing targeted Raman-label–SERS (RL-SERS)-nanotags which ensure a rapid, sensitive, and accurate multiplexed detection of clinically relevant breast cancer biomarkers, ER, PR, and HER2, in single tissue specimen by the marked signature Raman fingerprint resembling the corresponding biomarker [112].

Multiplex SERS staining of cancer biopsy sections using SERS nanotags was shown to visualize tumor heterogeneity. Murali and co-workers [112] recently demonstrated a novel spectroscopy-based approach for detecting multiple breast cancer biomarkers simultaneously in tumor tissue samples. The authors developed Raman-label SERS (RL-SERS) nanotags targeting three key prognostic markers—estrogen receptor (ER), progesterone receptor (PR), and human epidermal growth factor receptor 2 (HER2). The nanotags were constructed by attaching Raman reporter molecules and antibodies specific to each biomarker onto AuNPs. When applied to tumor samples, the RL-SERS nanotags bind to their target biomarkers, allowing sensitive detection through unique Raman spectral signatures. The authors first optimized the nanotags using breast cancer cell lines with known biomarker expression. They then applied the technique to archived formalin-fixed paraffin-embedded clinical tumor specimens to evaluate singleplex, duplex, and triplex detection capability (Figure 8). Using ratiometric analysis of the RL-SERS signatures, the sensitivity and specificity achieved was 95% and 92% for ER, 88% and 85% for PR, and 75% and 67% for HER2 in triplex detection. The nanotags also enabled semi-quantitative scoring of HER2 expression as 4+, 2+, or 1+, corresponding to fluorescence in situ hybridization. Large-area SERS imaging of 0.5–5 mm^2^ regions was performed to demonstrate feasibility for practical diagnostics. In another work, SERS could be used for the multiplexed molecular imaging of lung cancer biomarkers in vivo [113]. SERS utilizes AuNPs coated with Raman reporter molecules to generate bright, narrow spectral signatures that can be distinguished for multiplexing. In this proof-of-concept study, the authors targeted two antibodies (cetuximab and panitumumab) against different members of the epidermal growth factor receptor (EGFR) family, which is overexpressed in many cancers. They conjugated these antibodies to two different SERS nanoparticles carrying unique Raman reporters. Using a wide-field Raman imaging system, they first showed specific in vitro binding and detection of these anti-EGFR SERS nanoprobes in cultured cancer cells overexpressing EGFR. Significantly, for the first time, they then demonstrated simultaneous multiplexed imaging of both targeted SERS nanoprobes in vivo after topical application to EGFR-positive tumor xenografts in mice. This dual-receptor targeting approach compensated for nonspecific binding and improved tumor detection specificity.

For ultrasensitive analysis of liquid biopsy samples, multifunctional SERS platforms have been developed integrating microfluidics [114,115] and plasmonic substrates [116,117]. The immuno-capturing of exosomes followed by SERS using nanotags specific to exosomal protein markers allow for sensitive phenotypic profiling [118,119,120]. Portable lab-on-a-chip SERS devices enable point-of-care detection of cancer DNA and protein biomarkers from microliter volumes of patient blood, serum, saliva, or urine [121,122,123].

Several challenges need to be addressed for broader clinical translation of SERS cancer diagnostics. Fabrication protocols for reproducible and uniform SERS nanoparticles need to be standardized. The detection of biomarkers in complex media requires optimized surface coatings to prevent nonspecific adsorption. Improved multiplexing capabilities for high-throughput analysis should be enabled. The integration of machine learning methods for the automated analysis of SERS spectral data could aid in distinguishing normal versus disease states. Nevertheless, SERS has tremendous prospects for advancing cancer screening, diagnosis, surgical guidance, and treatment monitoring. The superlative sensitivity and molecular specificity offered by SERS combined with multiplexing abilities and increasing capabilities for real-time in vivo analysis position it as a transformative nanotechnology for next-generation precision cancer medicine.

## 4. Dark-Field Microscopy

**Figure 9:**
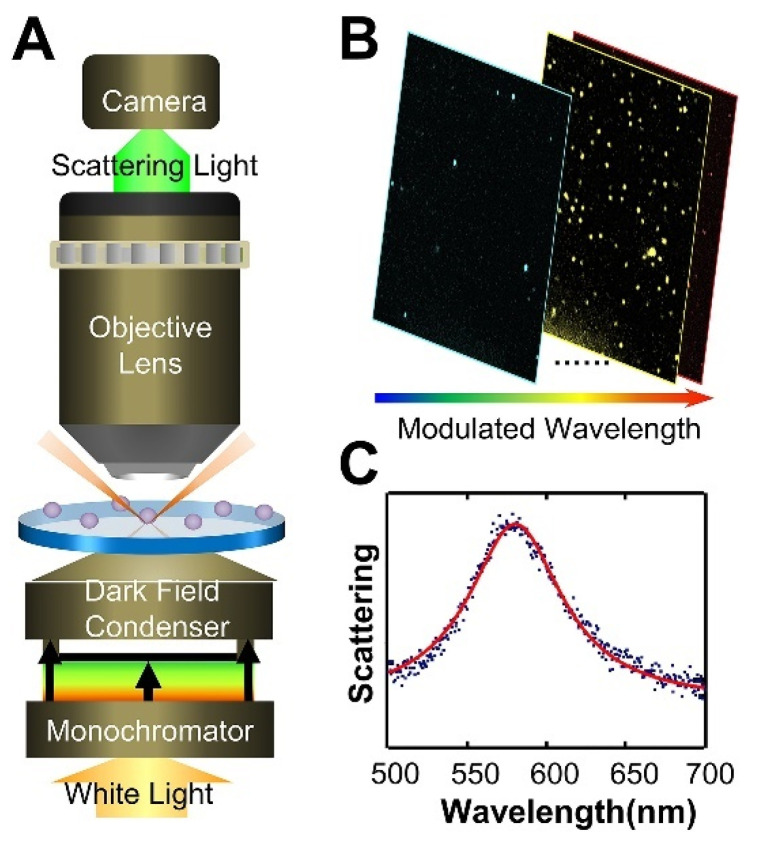
(**A**) Schematic graph of the home-built DFM. (**B**) The recorded images at different illumination wavelengths are combined into an image series. (**C**) Scattering spectra of one randomly selected particle [127].

DFM is an optical imaging technique that uses angled illumination to visualize light scattered from samples while blocking out background reflected light. This provides high-contrast images of nanoparticles and nanostructures [51,124]. Coupled with plasmonic nanoparticles, DFM offers a simple yet powerful platform for detecting and quantifying cancer biomarkers by eye or using spectral analysis [124,125]. In DFM, white light is focused on the sample from the periphery at oblique angles, while the directly reflected light is blocked by a light stop. Only the scattered light from the sample is collected by the objective lens for enhanced contrast imaging. Figure 9 shows the scheme of the DFM imaging system with incident wavelength modulation. Noble metal nanoparticles exhibit strong light scattering at their LSPR wavelength, which falls in the visible range. This resonant photon–electron coupling gives rise to brilliant colors and polarization-dependent scattering profiles. DFM illumination spectacularly excites the LSPR-derived scattering signal from individual nanoparticles and nanoparticle assemblies for high sensitivity detection [126].

**Figure 10:**
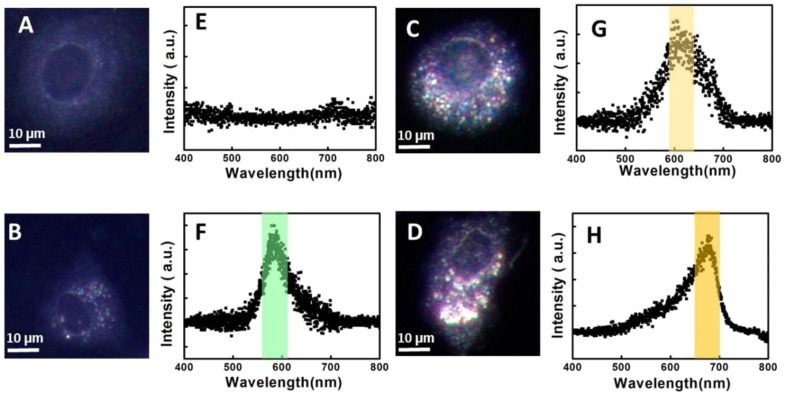
(**A**) DFM of blank SK-BR-3 cells. (**B**) DFM of SK-BR-3 cells incubated with anti-HER2/Au2−C≡C probes in a humidified atmosphere. (**C**) DFM of a single SK-BR-3 cell after further incubation with DNA1/Au1−N3 and anti-HER2/Au2−C≡C. (**D**) DFM of SK-BR-3 cell after RCA detection of HER2 in the SK-BR-3. (**E**–**H**) LSPR spectra of AuNP probes in (**A**–**D**), respectively [128].

Both solid plasmonic nanoparticles and hollow nanoshells have been applied as dark-field contrast agents. The scattering spectrum is highly dependent on nanoparticle size, shape, composition, and dielectric environment. This enables multiplexed detection using a cocktail of different nanoparticles tailored to distinct spectral signatures. Sophisticated synthesis methods allow for exquisite control over nanoparticle design to strategically tune the LSPR wavelength, intensity, and scattering directivity. For cancer diagnosis, DFM imaging of tissues, cytology samples, and purified biomarkers tagged with functionalized plasmonic nanoparticles have been demonstrated [31]. AuNRs have been used as optical probes and strand displacement amplification coupled with magnetic separation to achieve a detection limit of 71.22 fM of miRNA-21. The AuNRs are chemically etched in the presence of miRNA-21, resulting in a blue-shift and decrease in LSPR intensity that can be monitored by DFM. The strand displacement amplification provides high sensitivity, while the magnetic separation enables excellent specificity, even discriminating single nucleotide differences. The biosensor demonstrated a wide dynamic range from 0.1 to 10,000 pM miRNA-21. It was also successfully applied to detect miRNA-21 in human serum samples. Guo et al. [128] demonstrated a visual detection of proteins on live cell surfaces using DFM and specially designed AuNPs. The key innovation uses DFM to monitor a copper-catalyzed click reaction between azide and alkyne-modified AuNPs. When they react, it causes a red-shift in the LSPR peak of the AuNPs, detected as a color change from green to orange (Figure 10). To allow for the detection of HER2 proteins on cancer cells, they attached DNA and antibodies to two sets of AuNPs. One AuNP was functionalized with azide groups and DNA (Au1-N3). The other AuNP had alkyne groups and anti-HER2 antibodies (Au2-C≡C). The DNA on Au1-N3 allows for rolling circle amplification (RCA) to produce many complementary strands when triggered by HER2 proteins on breast cancer cells. This brings numerous Au1-N3 nanoparticles to the cell surface. There, they can undergo the click reaction with the Au2-C≡C nanoparticles already bound to HER2 proteins. The RCA amplification leads to much brighter LSPR signals detectable by DFM. Antibody-conjugated AuNPs have been used for the specific labeling and dark-field visualization of cancer biomarkers of carcinoembryonic antigen (CEA), PSA, and alpha fetoprotein (AFP) [51]. SeNPs conjugated to aptamers enable the sensitive detection of overexpressed nucleolin in cancer cells [129].

**Figure 11:**
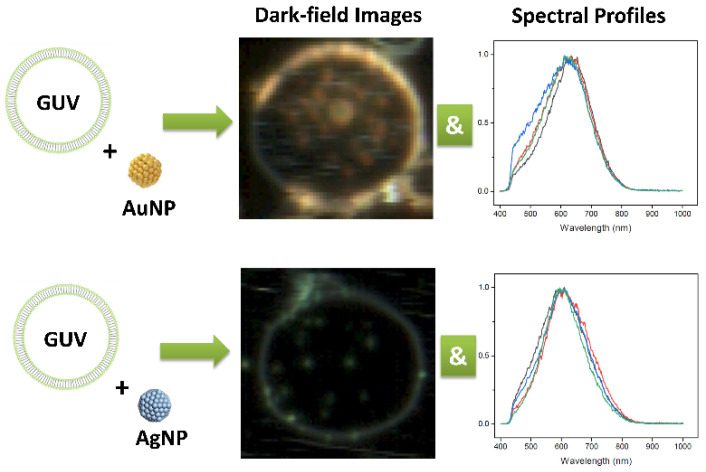
Probing interactions between AuNPs/AgNPs and GUVs using HSDFM [130].

Hyperspectral dark-field microscopy (HSDFM) combines spectroscopy and microscopy for both molecular and spatial information. Analyzing the full spectral data enables multiplexed quantification of multiple targets labeled with different colored nanoparticles. Advanced machine learning algorithms can be applied to spectral scatter fingerprints for automated classification of normal versus cancerous cells or tissues. For example, Bhat et al. [130] utilized HSDFM to investigate the interactions between 10 nm AuNPs or AgNPs and giant unilamellar vesicles (GUVs) made of the lipid dimyristoyl phosphatidylcholine (DMPC). GUVs were doped with varying concentrations of cholesterol to mimic mammalian cell membranes. The goal was to understand the physicochemical mechanisms governing AuNP and AgNP interactions with model cell membranes, which could inform their development for cancer targeting and treatment. As shown in Figure 11, AuNPs integrated into the lipid bilayer of the GUVs and formed a uniform golden-brown crust, suggesting AuNPs are highly disruptive to membrane integrity. In contrast, AgNPs interacted less disruptively, binding to the GUV surface as isolated particles or clusters with varied configurations. GUVs with 20% cholesterol content were optimal, exhibiting the greatest membrane integrity and lowest permeability. This aligns with other research showing that 20% cholesterol maximizes liquid-ordered domain formation critical for proper membrane function. The distinct mechanisms of AuNP and AgNP interaction with membranes could enable the selective targeting of cancerous vs. normal cells. AuNPs may permeabilize cancer cell membranes more readily due to aberrant lipid content, such as lower cholesterol. Cui et al. [131] demonstrated the use of HSDFM for analyzing individual cells. The authors developed a simple two-step method involving delipidation followed by refractive index matching to reduce intracellular light scattering and enhance the signal from nanoparticle probes. They found that lipid-rich organelles, such as endosomes and lysosomes, caused strong background scattering that obscured signals from plasmonic nanoparticles used to label targets. By first treating cells with isopropyl alcohol to extract lipids, these scattering structures were effectively removed. Subsequent immersion in ScaleA2 solution homogenized the refractive index throughout the cell to 1.39, further reducing scattering noise. Theoretical modeling showed optical clearing suppresses scattering from organelles while boosting signals from AuNPs, improving the signal-to-noise ratio. Experiments verified that this new clearing protocol significantly lowered scattering compared to other agents, enabling sensitive nanoparticle-based HSDFM imaging. The authors demonstrated high-resolution quantification and localization of receptors and mRNAs in single cancer cells. They also monitored intracellular AuNP growth dynamics with unprecedented spatiotemporal resolution. Simultaneously imaging multiple cell receptors can also be achieved using HSDFM [132]. The authors develop a multiplexed imaging system to analyze the immunophenotype of cells by detecting three commonly overexpressed cancer cell receptors—EGFR, IGF-1R, and HER-2. The authors chose AuNRs, Au nanospheres, and Ag nanospheres due to their distinct scattering spectra when imaged with hyperspectral microscopy. By conjugating antibodies specific to each receptor to one nanoparticle type, they created a system of molecular tags that could be imaged in one experiment. Experiments on cells with varying receptor expression demonstrated the specificity of each nanoparticle tag for its target receptor. Using single, double, and triple-labeled cells, the authors showed the ability to simultaneously detect the presence of the different receptors. This allowed for the determination of the immunophenotype of cell lines based on the combination of receptors detected.

**Figure 12:**
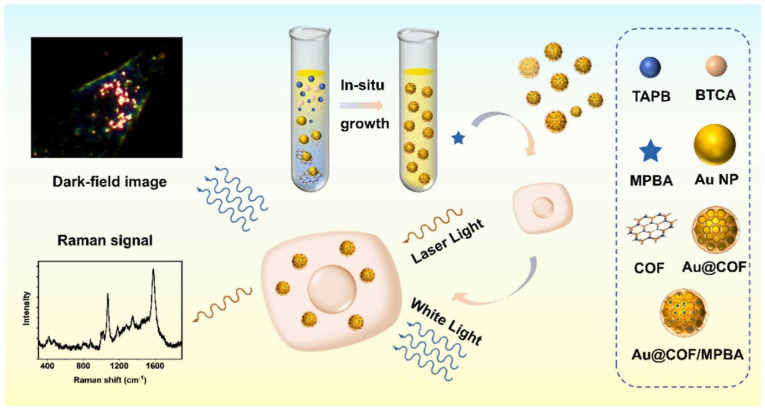
Schematic illustration of synthesis of Au@COF nanoparticle for detection of intracellular metabolite through dual-mode SERS/DFM technique [138].

DFM integrated with microfluidics and sample processing modules allow automated analysis of clinical blood, urine, sputum, and tissue samples [133,134,135]. Compact, field-portable DFM outfitted with multispectral imaging systems and telemedicine enabled smartphone interfaces are being developed [136]. These point-of-care dark-field diagnostics devices can provide rapid screening without requiring laboratory infrastructure or extensive training. Further integration of dark-field imaging with other optical and plasmon-enhanced techniques, such as SERS and photothermal imaging, could open up more applications in cancer diagnostics spanning the full range from molecular biochemistry to cellular pathology [137,138,139]. For example, Tan et al. [138] developed a core–shell-structured nanosensor (Au@COF) to detect intracellular hydrogen peroxide through a dual-mode technique combining SERS and DFM (Figure 12). The nanosensor consists of a AuNP core coated with a porous COF shell. The COF shell provides several advantages: it stabilizes the Au core, preconcentrates biomolecules for higher sensitivity, reduces cytotoxicity, and enables dispersion in cells. The authors optimized the shell thickness, with the thinnest shell of 3 nm still providing excellent SERS performance. For intracellular detection, the Au@COF nanosensor was incubated with living MCF-7 cells. SERS was used to sensitively detect a Raman reporter (MPBA) on the nanosensor that reacts with hydrogen peroxide. Simultaneously, DFM provided spatial imaging to locate the nanosensors and microregions in cells. Together, the dual-mode technique allowed for the real-time, in situ monitoring of hydrogen peroxide levels in single living cells. When cells were treated with different drugs, the Au@COF nanosensor successfully detected dynamic changes in intracellular hydrogen peroxide levels. With antimycin A to increase hydrogen peroxide, a six-fold enhancement of SERS signal was measured. In contrast, catalase caused SERS signal to decrease by 68%, indicating hydrogen peroxide consumption. DFM offers a versatile platform leveraging the light scattering properties of plasmonic nanoparticles for rapid visual identification as well as sensitive spectral quantification of cancer biomarkers in diverse samples. Ongoing advances in nanoparticle synthesis, surface functionalization, instrumentation, and automated image analysis will help propel dark-field diagnostic approaches towards widespread clinical implementation and point-of-care utility.

## 5. Photothermal Imaging

### 5.1. Concepts of Photothermal Imaging

Photothermal imaging is an emerging technique that holds great promise for sensitive visualization of cancer biomarkers by exploiting the efficient light-to-heat conversion properties of plasmonic noble metal nanoparticles [140]. Under laser irradiation at their LSPR wavelength, nanoparticles absorb optical energy and rapidly convert it to local heat. Monitoring the resultant temperature change enables thermal imaging contrast for biomarker mapping. Photothermal imaging offers deeper tissue penetration and minimal autofluorescence compared to conventional fluorescence and Raman techniques while achieving competitive sensitivity [141,142].

Various modalities of photothermal imaging have been implemented for cancer diagnosis. Photothermal microscopy uses a focused laser beam rastered across a sample containing LSPR nanoparticle labels while monitoring beam-induced temperature effects using a sensitive infrared detector [143]. Photothermal tomography and thermoacoustic imaging both utilize unfocused pulsed laser irradiation and ultrasound detection of acoustic waves generated by nanoparticle photothermal expansion for deep-tissue imaging [144]. Optical coherence tomography [145,146,147] and MRI [148,149,150] have also been combined with photothermal excitation of nanoparticles for hybrid imaging.

**Figure 13:**
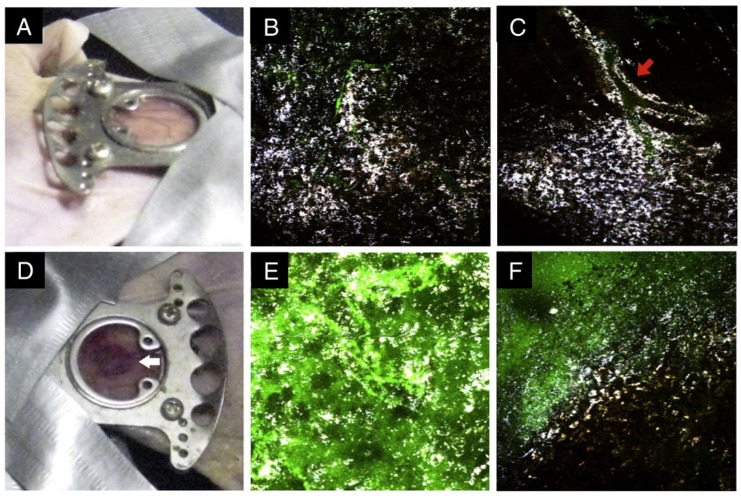
Photographs and multiphoton microscopy images 48 h after AuNSs infusion before (**top**) and after (**bottom**) the laser treatment. (**A**) Before the irradiation, the window appeared intact and uninflamed. (**B**) AuNSs (white color) scattered in the tissue with some remaining near the (**C**) perivascular space (red arrow). The green color from FITC-dextran delineates the blood vessels. (**D**) After the laser irradiation (785 nm 1.1 W/cm^2^, 10 min), a localized oozing of blood became visible (white arrow). I AuNSs distributed more randomly or into clusters. (**E**) Leakage of FITC-dextran into the tissue was appareIt in Ih” irr’diated spot (**F**) but not outside the spot [153].

Among noble metals, AuNPs exhibit the highest photothermal conversion efficiency due to their biocompatibility, chemical stability, and strong absorption cross-sections. The photothermal effect in Au nanomaterials can be strategically enhanced using shapes with sharp tips and edges, such as NRs [151,152], NSs [153,154], and nanoprisms [155,156,157] that concentrate electromagnetic fields for hotspot generation. For example, Yuan et al. [153] demonstrated the potential of AuNSs as efficient photothermal agents for cancer therapy through both in vitro and in vivo experiments. The authors synthesized surfactant-free AuNSs with NIR absorption tunable from 650 to 950 nm to match the tissue therapeutic window. The strong two-photon photoluminescence of AuNSs enables direct particle tracking under multiphoton microscopy. For in vitro tests, bare AuNSs incubated with SKBR3 breast cancer cells showed photothermal ablation within 5 min of 980 nm continuous wave laser irradiation at 15 W/cm^2^. For in vivo demonstration, PEGylated AuNSs were injected systemically into mice and allowed to accumulate and extravasate out of blood vessels over 48 h (Figure 13). In dorsal window chambers, localized photothermal ablation was achieved within 10 min of 785 nm laser irradiation at just 1.1 W/cm^2^. This lower irradiance is closer to the ANSI safety limit for skin (0.33 W/cm^2^ at 808 nm) than parameters used in other photothermal therapy studies. Post-irradiation, the window chambers showed blood oozing and tissue damage visible, both grossly and under multiphoton microscopy, validating the photothermal efficacy of AuNSs. Compared to previous in vivo work, the rapid ablation here, despite lower irradiance, highlights the superior photothermal performance of AuNSs. Their ability to damage tissue when injected systemically also illustrates potential for full-body cancer treatment. Hybrid core–shell nanostructures incorporating Pd [158], silica [159,160], Fe_3_O_4_ [161,162], or Cu_2−x_S [163] amplify photothermal signals and stabilize nanoparticles against degradation under laser irradiation.

### 5.2. Molecular Targeting Strategies

Molecular targeting strategies are critical for utilizing photothermal nanoparticles as agents for detecting cancer biomarkers. The conjugation of antibodies, aptamers, affibodies, small-molecules, and peptides to nanoparticle surfaces imparts specificity toward overexpressed tumor antigens, receptors, circulating factors, and molecular signatures of cancer cells [164,165,166,167,168,169]. Cancer cell membrane coating with nanoparticles enables photothermal detection even of poorly characterized targets based on differences in cell surface protein profiles between normal and cancerous cells [170]. Activatable photothermal probes, which are optically “switched on” by proteases, pH, or reactive oxygen species, provide sensing mechanisms attuned to the tumor microenvironment [171,172].

Photothermal imaging has been applied for the in vitro sensing of cancer cell cultures labeled with actively targeted plasmonic nanoparticles [173]. Photothermal flow cytometry techniques have been devised for quantification and longitudinal monitoring of rare circulating tumor cells in patient blood samples [174]. Multiplexed photothermal sensing is enabled using barcoded NRs with varying LSPR peaks excited by a tunable laser source [175]. For in vivo applications, photothermal nanoparticles coated with tumor-homing biomolecules injected intravenously have been used to highlight malignant lesions and identify tumor margins with high precision compared to conventional imaging [176].

**Figure 14:**
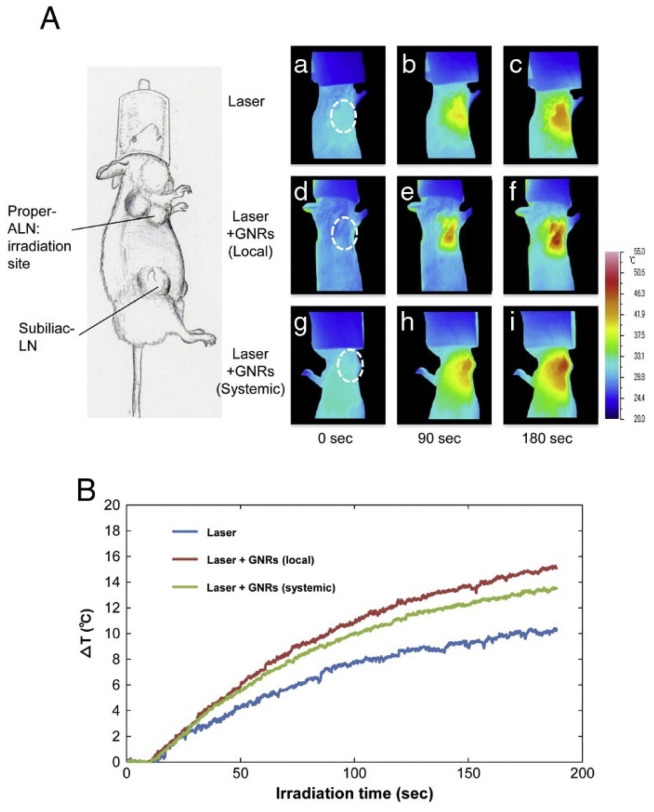
Laser irradiation of tumors in the proper lymph nodes. (**A**) Anatomical and thermographic images of tumors in the proper lymph nodes, irradiated on day 3. (a–c): laser light alone; (d–f): laser light with local AuNR delivery; (g–i): laser light with systemic GNR delivery. (a,d,g): irradiation time, 0 s; (b,e,h): irradiation time, 90 s; (c,f,i): irradiation time, 180 s. The dashed regions in (a), (d), and (g) indicate proper lymph node regions. (**B**) Increment in the maximum temperature, from ΔT = 0 (36 °C), against time, measured by thermography [178].

The real-time image-guided photothermal therapy of tumors has been demonstrated by leveraging the intrinsic thermal ablation capabilities of irradiated nanoparticles localized in cancerous tissues. Combining photothermal imaging and therapy (“theranostics”) in a single platform is a promising strategy for personalized nanomedicine approaches [177]. Photothermal nanoparticles are also being evaluated for aiding surgical resection and adjuvant treatment by marking lymph node metastases with near-infrared imaging and destroying residual cancer cells via hyperthermia [178]. Figure 14A shows representative thermal images of lymph nodes with tumors. The local temperature rise caused by laser irradiation was further increased when AuNRs were administered. The local injection of AuNRs into the tumor site was slightly more effective at raising the temperature than systemic GNR delivery. Figure 14B shows the maximum temperature increase from the baseline of 36 °C. After 3 min of laser irradiation alone, the temperature reached 46 °C. With the addition of AuNRs delivered locally, the temperature rose to 51 °C. Systemic AuNR delivery combined with laser irradiation resulted in a temperature of 50 °C after 3 min.

Ongoing efforts are focused on developing novel broadband-absorbing nanoparticle compositions for deeper tissue penetration, studying biomarker targeting efficiencies in physiologically relevant 3D culture models, and optimizing surface chemistry for minimal nonspecific uptake. The translation of photothermal nanoparticles to first-in-human clinical studies will require extensive evaluation of nanoparticle biodistribution, systemic toxicity, immunological effects, and long-term safety. Integrating machine learning with automated image analysis workflows can help extract subtle photothermal signatures from complex biological backgrounds. Overall, photothermal imaging and therapy using strategically engineered plasmonic nanotransducers provide an impactful nanotechnology platform for the sensitive and minimally invasive management of cancer.

## 6. Photoacoustic Imaging

### 6.1. Concepts of Photoacoustic Imaging

Photoacoustic imaging is an emerging biomedical imaging modality that offers deep-tissue penetration and high spatial resolution by detecting ultrasound waves generated through pulsed laser excitation of light-absorbing endogenous or exogenous contrast agents. In particular, plasmonic noble metal nanoparticles with strong optical absorption in the near-infrared window are ideal contrast agents for the photoacoustic imaging of cancer biomarkers [179,180]. The photoacoustic effect arises due to transient thermoelastic expansion induced by pulsed laser irradiation, which launches ultrasonic waves that can be detected using ultrasound transducers. As pulsed laser light illuminates photoabsorbing nanoparticles targeted to cancer biomarkers, they rapidly heat up and undergo thermal expansion, generating pressure waves [181]. The resulting ultrasound waves are detected by transducers placed around the tissue and processed using reconstruction algorithms into three-dimensional photoacoustic images.

Au and Ag nanocages [48], NRs [182], NSs [183], and nanoshells [184] with peak plasmon resonances in the 650–900 nm range are predominantly used as photoacoustic agents due to their biocompatibility, ease of synthesis, and high absorption coefficients. The photoacoustic signal strength depends on the nanoparticle optical absorption cross-section, concentration, and laser pulse energy. Silica coating enhances stability and allows for the conjugation of cancer-targeting moieties [185,186,187]. Multiplexing capabilities can be achieved using a cocktail of nanoparticles with distinct optical absorption spectra [188,189].

Molecularly targeted AuNPs have been used as contrast agents for visualizing tumors and metastases in small animal models using photoacoustic microscopy with spatial resolution better than 50 μm and a penetration depth of 3–5 cm [190]. Actively targeted photoacoustic nanoparticles are under development for detecting circulating tumor cells, cancer stem cells, and metastatic lesions by binding to overexpression cancer biomarkers [191,192,193]. Multi-wavelength photoacoustic imaging has been shown to differentiate nanoparticles accumulated in tumors versus normal tissues for sensitive and specific detection [194,195]. Handheld photoacoustic probes inserted into tissue cavities during endoscopic procedures enable sensing tumor margins in real-time [196]. Interventional photoacoustic needle probes capable of deep-tissue insertion have been designed for guiding biopsy and the localization of sentinel lymph nodes that require excision [197,198]. The functionalization of photoacoustic nanoparticles with therapeutic agents allows for theranostics for personalized treatment monitoring [199].

### 6.2. Photoacoustic Tomography (PAT)

**Figure 15:**
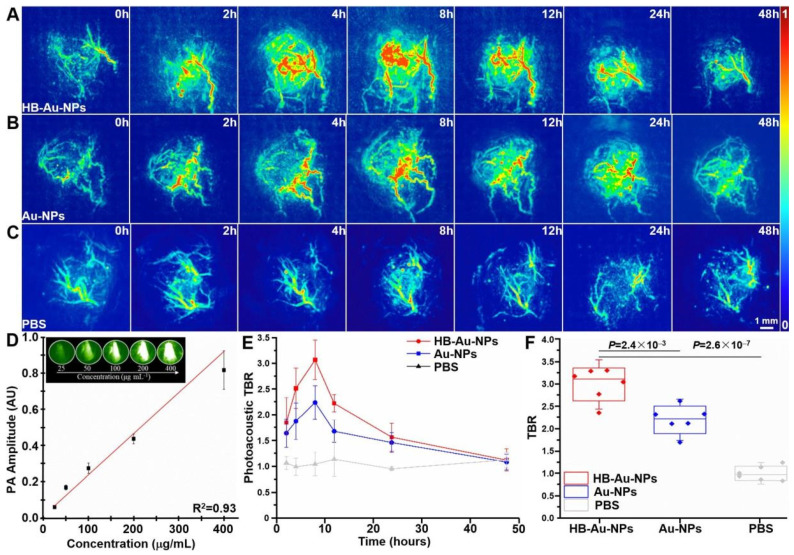
Photoacoustic tomography. In vivo images of human xenograft tumors implanted in mice are shown between 0 and 48 h post-injection of (**A**) HB-AuNPs, (**B**) AuNPs, and (**C**) PBS. (**D**) The in vitro photoacoustic intensity increases linearly with concentration of HB-AuNPs. (**E**) Quantified intensities from the tumors show a peak T/B ratio of 3.08 ± 0.37 and 2.27 ± 0.31 at 8 h post-injection for HB-AuNPs and AuNPs, respectively. (**F**) The mean value for HB-AuNPs is significantly greater than that for either AuNPs or PBS in *n* = six animals, *p* = 2.4 × 10^−3^, and 2.6 × 10^−7^, respectively, by unpaired *t*-test [201].

PAT systems using an ultrasonic transducer array for triangulated detection and tomographic reconstruction facilitate whole-body imaging [200]. PAT has been used to monitor tumor development and response to therapy longitudinally over weeks in vivo by tracking accumulations of circulating photoacoustic nanoparticles at tumor sites. For example, Chen et al. [201] developed ultra-small AuNPs coated with a thin layer of thiol-containing ligands and functionalized with a heterobivalent peptide to target the overexpression of EGFR and ErbB2 receptors in cancer cells. In vitro photoacoustic microscopy demonstrated significant uptake and contrast enhancement only in EGFR/ErbB2-positive cancer cells compared to benign cells after incubation with the nanoparticles. In vivo photoacoustic imaging in a xenograft model showed that the nanoparticles had optimal tumor uptake at 8 h post-injection, appearing as a strong photoacoustic signal in the tumor region. The signal peaked at around a five-fold increase compared to pre-injection levels and the surrounding tissue. There was clear tumor delineation in the photoacoustic images, with the tumor-to-background ratio reaching over two-fold higher than pre-injection. By 48 h, the photoacoustic signal had mostly cleared from the tumor and body (Figure 15). Importantly, CT imaging validated the specific tumor uptake and accumulation of the nanoparticles seen with photoacoustic imaging. Multispectral PAT can discriminate multiple targeted nanoparticle types simultaneously via their distinct optical absorption spectra for molecular multiplexing [202].

Ongoing efforts are focused on developing novel broadband-absorbing nanoparticles for deeper tissue imaging, optimizing surface properties for minimal non-specific uptake and long circulation half-lives, and conjugating cell-penetrating moieties to enable intracellular uptake and amplification of photoacoustic signals from sparse cancer biomarkers. Computational methods for analyzing complex multi-parametric photoacoustic datasets are being advanced. Overall, photoacoustic imaging enhanced by actively targeted plasmonic nanoparticles shows tremendous promise for non-invasive, high-resolution diagnostics and image-guided interventions for combating cancer.

## 7. Multiplexing and Point-of-Care Detection

**Figure 16:**
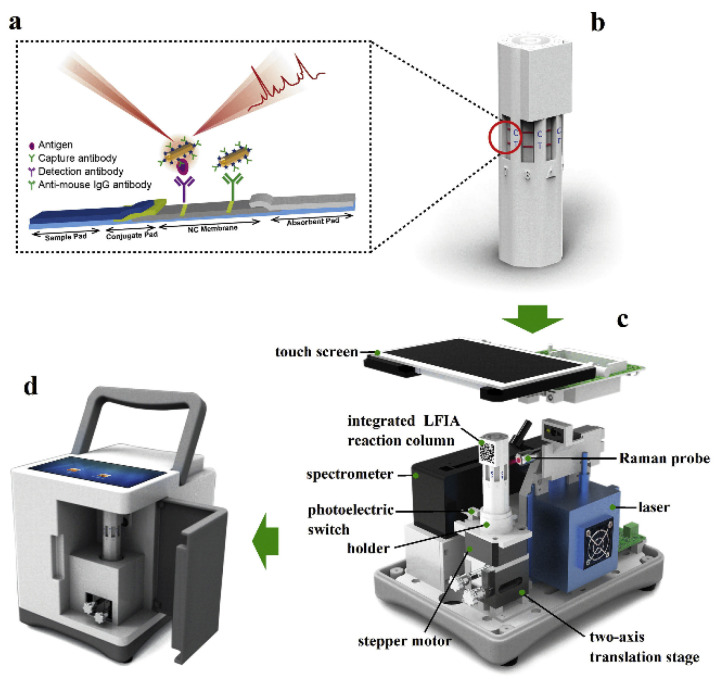
Schematic of portable SERS-based LFIA reader. (**a**) LFIA strip and (**b**) integrated reaction column. (**c**) Details of SERS-based LFIA reader and (**d**) the entire portable reader [205].

A key capability offered by LSPR nanotechnology that can greatly benefit cancer diagnostics is the multiplexed detection of biomarker panels for personalized risk profiling and therapy selection. As mentioned above, by tuning the LSPR peaks of different noble metal nanostructures, multiple biomarkers can be detected in parallel using a single sample. Integrating these multiplexing capabilities with microfluidics and miniaturized detectors can enable low-cost point-of-care diagnostic devices for non-invasive liquid biopsy analysis at the bedside. Multiplexing is advantageous because the abnormal expression of multiple genes and proteins provides a more accurate composite signature of cancerous states than a single biomarker. For example, low-density protein arrays spotted with different antibodies have been coupled with various LSPR nanoparticles encoded with distinct Raman reporters for quantifying up to ten protein cancer markers simultaneously [203]. Multiplexing capabilities of up to 100-fold have been demonstrated using barcode nanotags with finely tuned Raman shifts [204]. For example, Xiao et al. [205] developed a portable and multiplexed SERS-based lateral flow immunoassay (LFIA) reader integrated with a multichannel LFIA reaction column (Figure 16). This system can detect three cancer markers (AFP, CEA, and PSA). Similar SERS-encoded particles functionalized with different DNA probes allow for the parallel detection of multiple gene mutations or miRNA markers associated with cancer [206].

Dark-field microscopy coupled with hyperspectral analysis of distinct plasmonic nanoparticles targeting diverse cellular antigens has been applied for the consolidated molecular phenotyping of cancer cells and tissues [126]. Photoacoustic tomography using a mixture of tuned plasmonic nanostructures was able to simultaneously track and quantify circulating levels of different nanoparticles targeted to markers of tumor angiogenesis and hypoxia [207]. Such molecular multiplexing strategies using nano-engineered LSPR platforms provide a more nuanced, systems-level perspective of the complex interconnected cancer pathway circuitry compared to measuring single biomarkers in isolation. Quantifying biomarker co-expression patterns can improve predictive power for patient prognosis, disease staging, and therapeutic outcomes. Multiplexed assays allow for consolidating the detection of diverse biomarker types, such as proteins, nucleic acids, cells, and exosomes onto a single platform. This increases operational throughput while decreasing time, cost, and sample volume requirements compared to running separate tests.

However, to realize routine clinical utility, multiplexing capabilities need to be integrated with sample handling, processing, and analysis components into automated point-of-care devices usable at the patient bedside with minimal technical expertise. Lab-on-a-chip microfluidic systems incorporating LSPR nanosensors, on-chip sample purification modules, and optoelectronic components for signal transduction offer a promising route for realizing compact, portable devices for cancer screening and diagnosis [208].

LSPR-enhanced vertical flow assays on porous nitrocellulose pads integrated with microfluidics have been utilized for the sensitive quantification of protein biomarkers from microliter volumes [209]. Microfluidic mixing and incubation of LSPR nanotags with clinical samples improves reaction kinetics and amplification [210]. For decentralized testing, smartphone-based interfaces have been developed that can image and quantify colorimetric LSPR assays as well as acquire and analyze LSPR spectral data [211]. Paper-based microfluidic devices incorporating plasmonic nanoparticles as colorimetric reporters constitute low-cost alternatives to traditional lab-on-a-chip platforms [212,213].

Overall, the integration of LSPR nanoprobes with microscale device engineering and data analytic workflows can transform cancer diagnostics from expensive laboratory procedures requiring days to rapid near-patient tests providing actionable molecular information. Further technological innovation and multi-stakeholder coordination is critical in translating these technologies from academic laboratories to real-world clinical implementation.

## 8. Conclusions and Future Outlook

In this comprehensive review, we have surveyed the broad and dynamic landscape of LSPR nanotechnology for enabling the ultrasensitive optical detection of cancer biomarkers. The unique photonic properties of noble metal nanostructures arising from light-driven coherent electron oscillations confined at the nanoparticle surface provide a versatile platform for boosting intrinsically weak biomarker signals. Strategic engineering of nanoparticle size, shape, composition, and assembly geometry allows for the spectral tuning of LSPR peaks and optimization of near-field enhancement at hot spots. This underpins a diverse range of plasmon-enhanced optical sensing modalities, including surface-enhanced Raman spectroscopy, surface plasmon resonance, dark-field microscopy, photothermal imaging, and photoacoustic imaging.

We reviewed the latest developments in synthesizing innovative nanoparticle designs, engineering surface functionalization strategies, integrating LSPR with microfluidics, and multiplexing biomarker panels using barcoded nanotags. Ongoing research has leveraged LSPR advances to push the frontiers of detecting biomarkers such as circulating tumor cells, exosomes, miRNAs, mutated DNA, and cancer proteins at unprecedented femto–attomolar sensitivities. LSPR nanotechnology has also enabled the consolidation of cellular and molecular analyses along with in vivo imaging onto integrated platforms. These advances have positioned LSPR as a truly disruptive technology on the cusp of revolutionizing cancer diagnostics.

However, several key challenges remain to be addressed that currently constrain the widespread practical implementation of LSPR nanoplasmonics for cancer applications. The development of robust, reproducible, and scalable methods for fabricating uniform LSPR nanoprobes is essential. Systematic toxicity studies and investigations of long-term fate and clearance in vivo need to be performed thoroughly prior to clinical translation. Additionally, standardized surface functionalization protocols must be established to improve stability, targeting, and biocompatibility while minimizing non-specific interactions in complex biofluids. Detection schemes leveraging LSPR should be tested rigorously with real patient samples to evaluate clinical accuracy and reliability compared to established methods. User-friendly instrumentation, automated operation, and seamless data interpretation workflows need to be actualized for adoption beyond specialized labs. Regulatory guidelines will need to be navigated carefully as well.

Alternative SERS-active materials with improved biocompatibility would be valuable to address this limitation and enable clinical translation [214,215]. Carbon nanomaterials, such as graphene and carbon nanotubes, which are two-dimensional and one-dimensional carbon allotropes, exhibit strong Raman signals and can be functionalized with biomolecules for targeting. They also show good biocompatibility due to their chemical stability, inertness, and non-toxicity. However, their intrinsically lower enhancement factors compared to noble metals need to be improved through doping, patterning, and heterostructure formation. Transition metal dichalcogenides, such as MoS2 and WS2, which are layered semiconductors, have shown potential as SERS substrates and photothermal agents for cancer treatment. They benefit from having a layered structure similar to graphene but with a relatively large bandgap, enabling photoexcitation and plasmon-like resonances. Their biocompatibility still needs thorough evaluation, especially for nano-sized forms. Metal oxide nanoparticles, such as TiO_2_ and ZnO, have been investigated as SERS-active materials and photothermal/photodynamic therapy agents. They tend to be more biocompatible than noble metals due to their chemical stability and similarity to endogenous ions. However, their lower enhancement factors and broad absorption bands remain challenges for sensitive detection applications.

Tremendous opportunities exist for further advancing LSPR nanodiagnostics through synergistic convergence with other emerging technologies. Integration with microfluidics, point-of-care platforms, wearables, and smartphone interfaces can enable widespread decentralized deployment. Coupling with advanced multiplexing assays, optical traps, machine learning, and artificial intelligence algorithms can enhance capabilities. Hybridization with complementary techniques, such as mass spectrometry, sequencing, Raman spectroscopy, and ultrasound, can augment the strengths of LSPR platforms. The incorporation of therapeutic modules for combined diagnosis and therapy (“theranostics”) can provide new avenues for personalized nanomedicine.

In summary, building on the explosive growth over the past decade, LSPR nanotechnologies are poised to transform cancer diagnostics in the coming years through synergistic innovation across multiple disciplines. This holds promise for non-invasive early detection, real-time molecular profiling of tumors, and guiding therapeutic interventions—all geared towards dramatically improving clinical outcomes for cancer patients. The prospects are compelling for plasmonic nanosensing to help realize the vision of next-generation precision oncology care.

## Figures and Tables

**Table 1:** Definitions and calculation/measurement methods for key terminology in evaluating biosensing performance.

Term	Definition	Typical Calculation/Measurement
Sensitivity	The ability of a biosensor to detect small changes in the concentration of an analyte. It is expressed as the slope of the calibration curve or the ratio of the change in sensor response to the change in analyte concentration. A higher sensitivity indicates a more responsive biosensor.	Slope of the linear region of calibration curve (signal vs. analyte concentration)
Limit of detection (LOD)	The lowest concentration of an analyte that can be reliably detected by a biosensor. It is an important figure of merit, with a lower LOD indicating a more sensitive biosensor.	Concentration corresponding to signal equivalent to blank mean + 3 × standard deviation of blank
Linear detection range	Concentration range over which the biosensor response is directly proportional to the analyte concentration. Wider linear range allows for quantifying analytes over a broader concentration span.	Concentration range over which calibration curve is linear (R2~1)
Selectivity	The ability of a biosensor to respond specifically to the target analyte in the presence of other interfering species. A highly selective biosensor has minimal cross-reactivity with non-target analytes.	Measured by comparing the sensor response to the target analyte versus non-target analytes at the same concentration.
Quality factor (Q factor)	A measure of the sharpness of the resonance peak of a plasmonic nanostructure. A higher Q factor indicates a narrower resonance peak and a more sensitive response to changes in the local environment.	Q = λres/Δλ, where λres = LSPR peak wavelength, Δλ = full width at half max
EF	The ratio of the sensor response (e.g., Raman scattering intensity) with a plasmonic nanostructure to the response without it. A higher EF indicates greater signal amplification by the plasmonic nanostructure.	EF = Ienhanced/Ioriginal, where I = intensity of enhanced and original signals
Reproducibility	Variability in response when the same experiment is repeated multiple times under same conditions, expressed using relative standard deviation. Lower variability indicates more consistent performance.	Standard deviation/mean of repeated measurements under same conditions
Response time	Time required to achieve stable sensor response after analyte introduction. Shorter response time enables faster detection.	Time from analyte introduction to achievement of stable sensor signal

## Data Availability

No new data were created or analyzed in this study. Data sharing is not applicable to this article.

## References

[1] Cao W., Chen H.-D., Yu Y.-W., Li N., Chen W.-Q. (2021). Changing Profiles of Cancer Burden Worldwide and in China: A Secondary Analysis of the Global Cancer Statistics 2020. Chin. Med. J..

[2] Crosby D., Bhatia S., Brindle K.M., Coussens L.M., Dive C., Emberton M., Esener S., Fitzgerald R.C., Gambhir S.S., Kuhn P. (2022). Early Detection of Cancer. Science.

[3] Van Der Pol Y., Mouliere F. (2019). Toward the Early Detection of Cancer by Decoding the Epigenetic and Environmental Fingerprints of Cell-Free DNA. Cancer Cell.

[4] Arshad F., Nabi F., Iqbal S., Khan R.H. (2022). Applications of Graphene-Based Electrochemical and Optical Biosensors in Early Detection of Cancer Biomarkers. Colloids Surf. B Biointerfaces.

[5] Chen X., Gole J., Gore A., He Q., Lu M., Min J., Yuan Z., Yang X., Jiang Y., Zhang T. (2020). Non-Invasive Early Detection of Cancer Four Years before Conventional Diagnosis Using a Blood Test. Nat. Commun..

[6] Pashayan N., Pharoah P.D. (2020). The Challenge of Early Detection in Cancer. Science.

[7] Abbasi Kajani A., Haghjooy Javanmard S., Asadnia M., Razmjou A. (2021). Recent Advances in Nanomaterials Development for Nanomedicine and Cancer. ACS Appl. Bio Mater..

[8] Wang H., Wu T., Li M., Tao Y. (2021). Recent Advances in Nanomaterials for Colorimetric Cancer Detection. J. Mater. Chem. B.

[9] Fu L., Zheng Y., Li X., Liu X., Lin C.-T., Karimi-Maleh H. (2023). Strategies and Applications of Graphene and Its Derivatives-Based Electrochemical Sensors in Cancer Diagnosis. Molecules.

[10] Karimi F., Karimi-Maleh H., Rouhi J., Zare N., Karaman C., Baghayeri M., Fu L., Rostamnia S., Dragoi E.N., Ayati A. (2023). Revolutionizing Cancer Monitoring with Carbon-Based Electrochemical Biosensors. Environ. Res..

[11] Karimi-Maleh H., Alizadeh M., Orooji Y., Karimi F., Baghayeri M., Rouhi J., Tajik S., Beitollahi H., Agarwal S., Gupta V.K. (2021). Guanine-Based DNA Biosensor Amplified with Pt/SWCNTs Nanocomposite as Analytical Tool for Nanomolar Determination of Daunorubicin as an Anticancer Drug: A Docking/Experimental Investigation. Ind. Eng. Chem. Res..

[12] Karimi-Maleh H., Khataee A., Karimi F., Baghayeri M., Fu L., Rouhi J., Karaman C., Karaman O., Boukherroub R. (2022). A Green and Sensitive Guanine-Based DNA Biosensor for Idarubicin Anticancer Monitoring in Biological Samples: A Simple and Fast Strategy for Control of Health Quality in Chemotherapy Procedure Confirmed by Docking Investigation. Chemosphere.

[13] Jian C., Zhang J., He W., Ma X. (2021). Au-Al Intermetallic Compounds: A Series of More Efficient LSPR Materials for Hot Carriers-Based Applications than Noble Metal Au. Nano Energy.

[14] He W., Huang X., Ma X., Zhang J. (2022). Significant Temperature Effect on the LSPR Properties of Noble Metal Nanoparticles. J. Opt..

[15] Li J., Zhang Y., Huang Y., Luo B., Jing L., Jing D. (2022). Noble-Metal Free Plasmonic Nanomaterials for Enhanced Photocatalytic Applications—A Review. Nano Res..

[16] Fu L., Zhu D., Yu A. (2015). Galvanic Replacement Synthesis of Silver Dendrites-Reduced Graphene Oxide Composites and Their Surface-Enhanced Raman Scattering Characteristics. Spectrochim. Acta Part A Mol. Biomol. Spectrosc..

[17] Li J., Su W., Chen F., Fu L., Ding S., Song K., Huang X., Zhang L. (2019). Atypical Defect-Mediated Photoluminescence and Resonance Raman Spectroscopy of Monolayer WS2. J. Phys. Chem. C.

[18] Zhou Q., Jin M., Wu W., Fu L., Yin C., Karimi-Maleh H. (2022). Graphene-Based Surface-Enhanced Raman Scattering (SERS) Sensing: Bibliometrics Based Analysis and Review. Chemosensors.

[19] Fu Y., Zeng G., Lai C., Huang D., Qin L., Yi H., Liu X., Zhang M., Li B., Liu S. (2020). Hybrid Architectures Based on Noble Metals and Carbon-Based Dots Nanomaterials: A Review of Recent Progress in Synthesis and Applications. Chem. Eng. J..

[20] Ma X., He S., Qiu B., Luo F., Guo L., Lin Z. (2019). Noble Metal Nanoparticle-Based Multicolor Immunoassays: An Approach toward Visual Quantification of the Analytes with the Naked Eye. ACS Sens..

[21] Hu Y., Zhang B.Y., Haque F., Ren G., Ou J.Z. (2022). Plasmonic Metal Oxides and Their Biological Applications. Mater. Horiz..

[22] Karimi-Maleh H., Liu Y., Li Z., Darabi R., Orooji Y., Karaman C., Karimi F., Baghayeri M., Rouhi J., Fu L. (2023). Calf Thymus Ds-DNA Intercalation with Pendimethalin Herbicide at the Surface of ZIF-8/Co/rGO/C3N4/Ds-DNA/SPCE; A Bio-Sensing Approach for Pendimethalin Quantification Confirmed by Molecular Docking Study. Chemosphere.

[23] Khademalrasool M., Farbod M., Talebzadeh M.D. (2021). Near-Field and Far-Field Optical Properties of Silver Nanospheres: Theoretical and Experimental Investigations of the Size, Shape, Dielectric Environment, and Composition Effects. Prot. Met. Phys. Chem. Surf..

[24] Li J.-J., Qin Q.-X., Weng G.-J., Zhu J., Zhao J.-W. (2022). Improve the Hole Size–Dependent Refractive Index Sensitivity of Au–Ag Nanocages by Tuning the Alloy Composition. Plasmonics.

[25] Ferhan A.R., Jackman J.A., Park J.H., Cho N.-J., Kim D.-H. (2018). Nanoplasmonic Sensors for Detecting Circulating Cancer Biomarkers. Adv. Drug Deliv. Rev..

[26] Bellassai N., D’Agata R., Jungbluth V., Spoto G. (2019). Surface Plasmon Resonance for Biomarker Detection: Advances in Non-Invasive Cancer Diagnosis. Front. Chem..

[27] Hong Y., Huh Y.-M., Yoon D.S., Yang J. (2012). Nanobiosensors Based on Localized Surface Plasmon Resonance for Biomarker Detection. J. Nanomater..

[28] Hwang W.S., Sim S.J. (2011). A Strategy for the Ultrasensitive Detection of Cancer Biomarkers Based on the LSPR Response of a Single AuNP. J. Nanosci. Nanotechnol..

[29] Lenzi E., Jimenez de Aberasturi D., Liz-Marzán L.M. (2019). Surface-Enhanced Raman Scattering Tags for Three-Dimensional Bioimaging and Biomarker Detection. ACS Sens..

[30] Azzouz A., Hejji L., Kim K.-H., Kukkar D., Souhail B., Bhardwaj N., Brown R.J.C., Zhang W. (2022). Advances in Surface Plasmon Resonance–Based Biosensor Technologies for Cancer Biomarker Detection. Biosens. Bioelectron..

[31] Zhang Q., Yan H.H., Ru C., Zhu F., Zou H.Y., Gao P.F., Huang C.Z., Wang J. (2022). Plasmonic Biosensor for the Highly Sensitive Detection of microRNA-21 via the Chemical Etching of Gold Nanorods under a Dark-Field Microscope. Biosens. Bioelectron..

[32] Wang Z., Wang M., Wang X., Hao Z., Han S., Wang T., Zhang H. (2023). Photothermal-Based Nanomaterials and Photothermal-Sensing: An Overview. Biosens. Bioelectron..

[33] Zhao Z., Swartchick C.B., Chan J. (2022). Targeted Contrast Agents and Activatable Probes for Photoacoustic Imaging of Cancer. Chem. Soc. Rev..

[34] Sousa D.A., Carneiro M., Ferreira D., Moreira F.T.C., Sales M.G.F., Rodrigues L.R. (2022). Recent Advances in the Selection of Cancer-Specific Aptamers for the Development of Biosensors. Curr. Med. Chem..

[35] Choi J.-H., Lee J.-H., Son J., Choi J.-W. (2020). Noble Metal-Assisted Surface Plasmon Resonance Immunosensors. Sensors.

[36] Cheng L., Zhang Z., Zuo D., Zhu W., Zhang J., Zeng Q., Yang D., Li M., Zhao Y. (2018). Ultrasensitive Detection of Serum MicroRNA Using Branched DNA-Based SERS Platform Combining Simultaneous Detection of α-Fetoprotein for Early Diagnosis of Liver Cancer. ACS Appl. Mater. Interfaces.

[37] Bayda S., Hadla M., Palazzolo S., Riello P., Corona G., Toffoli G., Rizzolio F. (2018). Inorganic Nanoparticles for Cancer Therapy: A Transition from Lab to Clinic. Curr. Med. Chem..

[38] Yao J., Wang L.V. (2018). Recent Progress in Photoacoustic Molecular Imaging. Curr. Opin. Chem. Biol..

[39] Gong T., Olivo M., Dinish U.S., Goh D., Kong K.V., Yong K.-T. (2013). Engineering Bioconjugated Gold Nanospheres and Gold Nanorods as Label-Free Plasmon Scattering Probes for Ultrasensitive Multiplex Dark-Field Imaging of Cancer Cells. J. Biomed. Nanotechnol..

[40] Pires N.M.M., Dong T., Hanke U., Hoivik N. (2014). Recent Developments in Optical Detection Technologies in Lab-on-a-Chip Devices for Biosensing Applications. Sensors.

[41] Tai J., Fan S., Ding S., Ren L. (2022). Gold Nanoparticles Based Optical Biosensors for Cancer Biomarker Proteins: A Review of the Current Practices. Front. Bioeng. Biotechnol..

[42] Mulyanti B., Nugroho H.S., Wulandari C., Rahmawati Y., Hasanah L., Hamidah I., Pawinanto R.E., Majlis B.Y. (2022). SPR-Based Sensor for the Early Detection or Monitoring of Kidney Problems. Int. J. Biomater..

[43] Mayer K.M., Hafner J.H. (2011). Localized Surface Plasmon Resonance Sensors. Chem. Rev..

[44] Bhatia P., Verma S.S. (2023). Enhancement of LSPR Properties of Temperature-Dependent Gold Nanoparticles. Mater. Today Proc..

[45] Amirjani A., Firouzi F., Haghshenas D.F. (2020). Predicting the Size of Silver Nanoparticles from Their Optical Properties. Plasmonics.

[46] He M.-Q., Yu Y.-L., Wang J.-H. (2020). Biomolecule-Tailored Assembly and Morphology of Gold Nanoparticles for LSPR Applications. Nano Today.

[47] Shabaninezhad M., Ramakrishna G. (2019). Theoretical Investigation of Size, Shape, and Aspect Ratio Effect on the LSPR Sensitivity of Hollow-Gold Nanoshells. J. Chem. Phys..

[48] Li W., Brown P.K., Wang L.V., Xia Y. (2011). Gold Nanocages as Contrast Agents for Photoacoustic Imaging. Contrast Media Mol. Imaging.

[49] Auner G.W., Koya S.K., Huang C., Broadbent B., Trexler M., Auner Z., Elias A., Mehne K.C., Brusatori M.A. (2018). Applications of Raman Spectroscopy in Cancer Diagnosis. Cancer Metastasis Rev..

[50] Qian X.-M., Nie S.M. (2008). Single-Molecule and Single-Nanoparticle SERS: From Fundamental Mechanisms to Biomedical Applications. Chem. Soc. Rev..

[51] Poon C.-Y., Wei L., Xu Y., Chen B., Xiao L., Li H.-W. (2016). Quantification of Cancer Biomarkers in Serum Using Scattering-Based Quantitative Single Particle Intensity Measurement with a Dark-Field Microscope. Anal. Chem..

[52] Liu Y., Bhattarai P., Dai Z., Chen X. (2019). Photothermal Therapy and Photoacoustic Imaging via Nanotheranostics in Fighting Cancer. Chem. Soc. Rev..

[53] Navas M.P., Soni R.K. (2015). Laser-Generated Bimetallic Ag-Au and Ag-Cu Core-Shell Nanoparticles for Refractive Index Sensing. Plasmonics.

[54] Bansal A., Sekhon J.S., Verma S.S. (2014). Scattering Efficiency and LSPR Tunability of Bimetallic Ag, Au, and Cu Nanoparticles. Plasmonics.

[55] Ma J., Liu X., Wang R., Zhang J., Jiang P., Wang Y., Tu G. (2020). Bimetallic Core–Shell Nanostars with Tunable Surface Plasmon Resonance for Surface-Enhanced Raman Scattering. ACS Appl. Nano Mater..

[56] Mahmud S., Satter S.S., Singh A.K., Rahman M.M., Mollah M.Y.A., Susan M.A.B.H. (2019). Tailored Engineering of Bimetallic Plasmonic Au@Ag Core@Shell Nanoparticles. ACS Omega.

[57] Lismont M., Páez C.A., Dreesen L. (2015). A One-Step Short-Time Synthesis of Ag@SiO_2_ Core–Shell Nanoparticles. J. Colloid Interface Sci..

[58] Chen J.-J., Wu J.C.S., Wu P.C., Tsai D.P. (2012). Improved Photocatalytic Activity of Shell-Isolated Plasmonic Photocatalyst Au@SiO2/TiO2 by Promoted LSPR. J. Phys. Chem. C.

[59] Ling J., Zhi Huang C. (2010). Energy Transfer with Gold Nanoparticles for Analytical Applications in the Fields of Biochemical and Pharmaceutical Sciences. Anal. Methods.

[60] Yan Y., Chen J.I.L., Ginger D.S. (2012). Photoswitchable Oligonucleotide-Modified Gold Nanoparticles: Controlling Hybridization Stringency with Photon Dose. Nano Lett..

[61] Lee S.-W., Lee K.-S., Ahn J., Lee J.-J., Kim M.-G., Shin Y.-B. (2011). Highly Sensitive Biosensing Using Arrays of Plasmonic Au Nanodisks Realized by Nanoimprint Lithography. ACS Nano.

[62] Quilis N.G., Lequeux M., Venugopalan P., Khan I., Knoll W., Boujday S., de la Chapelle M.L., Dostalek J. (2018). Tunable Laser Interference Lithography Preparation of Plasmonic Nanoparticle Arrays Tailored for SERS. Nanoscale.

[63] Piantanida L., Naumenko D., Torelli E., Marini M., Bauer D.M., Fruk L., Firrao G., Lazzarino M. (2015). Plasmon Resonance Tuning Using DNA Origami Actuation. Chem. Commun..

[64] Dong J., Zhou Y., Pan J., Zhou C., Wang Q. (2021). Assembling Gold Nanobipyramids into Chiral Plasmonic Nanostructures with DNA Origami. Chem. Commun..

[65] Lu M., Peng W., Lin M., Wang F., Zhang Y. (2021). Gold Nanoparticle-Enhanced Detection of DNA Hybridization by a Block Copolymer-Templating Fiber-Optic Localized Surface Plasmon Resonance Biosensor. Nanomaterials.

[66] Singh L., Singh R., Zhang B., Cheng S., Kumar Kaushik B., Kumar S. (2019). LSPR Based Uric Acid Sensor Using Graphene Oxide and Gold Nanoparticles Functionalized Tapered Fiber. Opt. Fiber Technol..

[67] Zangeneh Kamali K., Pandikumar A., Jayabal S., Ramaraj R., Lim H.N., Ong B.H., Bien C.S.D., Kee Y.Y., Huang N.M. (2016). Amalgamation Based Optical and Colorimetric Sensing of Mercury(II) Ions with Silver@graphene Oxide Nanocomposite Materials. Microchim. Acta.

[68] Pons T., Medintz I.L., Sapsford K.E., Higashiya S., Grimes A.F., English D.S., Mattoussi H. (2007). On the Quenching of Semiconductor Quantum Dot Photoluminescence by Proximal Gold Nanoparticles. Nano Lett..

[69] Liu J., Detrembleur C., Mornet S., Jérôme C., Duguet E. (2015). Design of Hybrid Nanovehicles for Remotely Triggered Drug Release: An Overview. J. Mater. Chem. B.

[70] Raza A., Hayat U., Rasheed T., Bilal M., Iqbal H.M.N. (2019). “Smart” Materials-Based near-Infrared Light-Responsive Drug Delivery Systems for Cancer Treatment: A Review. J. Mater. Res. Technol..

[71] Limonov M.F., Rybin M.V., Poddubny A.N., Kivshar Y.S. (2017). Fano Resonances in Photonics. Nat. Photon..

[72] Liu Z., Ye J. (2016). Highly Controllable Double Fano Resonances in Plasmonic Metasurfaces. Nanoscale.

[73] Du M., Shen Z. (2021). Enhanced and Tunable Double Fano Resonances in Plasmonic Metasurfaces with Nanoring Dimers. J. Phys. D Appl. Phys..

[74] Campione S., Guclu C., Ragan R., Capolino F. (2014). Enhanced Magnetic and Electric Fields via Fano Resonances in Metasurfaces of Circular Clusters of Plasmonic Nanoparticles. ACS Photonics.

[75] Liang Y., Koshelev K., Zhang F., Lin H., Lin S., Wu J., Jia B., Kivshar Y. (2020). Bound States in the Continuum in Anisotropic Plasmonic Metasurfaces. Nano Lett..

[76] Tang Y., Liang Y., Yao J., Chen M.K., Lin S., Wang Z., Zhang J., Huang X.G., Yu C., Tsai D.P. (2023). Chiral Bound States in the Continuum in Plasmonic Metasurfaces. Laser Photonics Rev..

[77] Du W., Wen X., Gérard D., Qiu C.-W., Xiong Q. (2019). Chiral Plasmonics and Enhanced Chiral Light-Matter Interactions. Sci. China Phys. Mech. Astron..

[78] Bochenkov V.E., Shabatina T.I. (2018). Chiral Plasmonic Biosensors. Biosensors.

[79] Wu X., Xu L., Liu L., Ma W., Yin H., Kuang H., Wang L., Xu C., Kotov N.A. (2013). Unexpected Chirality of Nanoparticle Dimers and Ultrasensitive Chiroplasmonic Bioanalysis. J. Am. Chem. Soc..

[80] Nagarajan A., Panchanathan A.P., Chelliah P., Satoh H., Inokawa H. (2019). Optimization of Electric Field Enhancement of Ag@SiO2 Trimer Nanospheres by Finite Difference Time Domain Method. Appl. Surf. Sci..

[81] Bell S.E.J., Charron G., Cortés E., Kneipp J., de la Chapelle M.L., Langer J., Procházka M., Tran V., Schlücker S. (2020). Towards Reliable and Quantitative Surface-Enhanced Raman Scattering (SERS): From Key Parameters to Good Analytical Practice. Angew. Chem. Int. Ed..

[82] Litti L., Meneghetti M. (2019). Predictions on the SERS Enhancement Factor of Gold Nanosphere Aggregate Samples. Phys. Chem. Chem. Phys..

[83] Jung Seo M., Wan Kim G., Vuka Tsalu P., Woo Moon S., Won Ha J. (2020). Role of Chemical Interface Damping for Tuning Chemical Enhancement in Resonance Surface-Enhanced Raman Scattering of Plasmonic Gold Nanorods. Nanoscale Horiz..

[84] Ji C., Lu J., Shan B., Li F., Zhao X., Yu J., Xu S., Man B., Zhang C., Li Z. (2022). The Origin of Mo2C Films for Surface-Enhanced Raman Scattering Analysis: Electromagnetic or Chemical Enhancement?. J. Phys. Chem. Lett..

[85] Wang H.-N., Crawford B.M., Norton S.J., Vo-Dinh T. (2019). Direct and Label-Free Detection of MicroRNA Cancer Biomarkers Using SERS-Based Plasmonic Coupling Interference (PCI) Nanoprobes. J. Phys. Chem. B.

[86] Yang T., Guo X., Wu Y., Wang H., Fu S., Wen Y., Yang H. (2014). Facile and Label-Free Detection of Lung Cancer Biomarker in Urine by Magnetically Assisted Surface-Enhanced Raman Scattering. ACS Appl. Mater. Interfaces.

[87] He S., Kyaw Y.M.E., Tan E.K.M., Bekale L., Kang M.W.C., Kim S.S.-Y., Tan I., Lam K.-P., Kah J.C.Y. (2018). Quantitative and Label-Free Detection of Protein Kinase a Activity Based on Surface-Enhanced Raman Spectroscopy with Gold Nanostars. Anal. Chem..

[88] Beyene A.B., Hwang B.J., Tegegne W.A., Wang J.-S., Tsai H.-C., Su W.-N. (2020). Reliable and Sensitive Detection of Pancreatic Cancer Marker by Gold Nanoflower-Based SERS Mapping Immunoassay. Microchem. J..

[89] Verdin A., Malherbe C., Müller W.H., Bertrand V., Eppe G. (2020). Multiplex Micro-SERS Imaging of Cancer-Related Markers in Cells and Tissues Using Poly(Allylamine)-Coated Au@Ag Nanoprobes. Anal. Bioanal. Chem..

[90] Fălămaș A., Rotaru H., Hedeșiu M. (2020). Surface-Enhanced Raman Spectroscopy (SERS) Investigations of Saliva for Oral Cancer Diagnosis. Lasers Med. Sci..

[91] Bamrungsap S., Treetong A., Apiwat C., Wuttikhun T., Dharakul T. (2016). SERS-Fluorescence Dual Mode Nanotags for Cervical Cancer Detection Using Aptamers Conjugated to Gold-Silver Nanorods. Microchim. Acta.

[92] Wang X.-P., Walkenfort B., König M., König L., Kasimir-Bauer S., Schlücker S. (2017). Fast and Reproducible iSERS Microscopy of Single HER2-Positive Breast Cancer Cells Using Gold Nanostars as SERS Nanotags. Faraday Discuss..

[93] Eom G., Hwang A., Kim H., Moon J., Kang H., Jung J., Lim E.-K., Jeong J., Park H.G., Kang T. (2021). Ultrasensitive Detection of Ovarian Cancer Biomarker Using Au Nanoplate SERS Immunoassay. BioChip J..

[94] Hu C., Shen J., Yan J., Zhong J., Qin W., Liu R., Aldalbahi A., Zuo X., Song S., Fan C. (2016). Highly Narrow Nanogap-Containing Au@Au Core–Shell SERS Nanoparticles: Size-Dependent Raman Enhancement and Applications in Cancer Cell Imaging. Nanoscale.

[95] Chen K.-H., Pan M.-J., Jargalsaikhan Z., Ishdorj T.-O., Tseng F.-G. (2020). Development of Surface-Enhanced Raman Scattering (SERS)-Based Surface-Corrugated Nanopillars for Biomolecular Detection of Colorectal Cancer. Biosensors.

[96] Omar G., Abd Ellah R.G., Elzayat M.M.Y., Afifi G., Imam H. (2024). Superior Removal of Hazardous Dye Using Ag/Au Core–Shell Nanoparticles Prepared by Laser Ablation. Opt. Laser Technol..

[97] Awiaz G., Lin J., Wu A. (2023). Recent Advances of Au@Ag Core–Shell SERS-Based Biosensors. Exploration.

[98] Dinish U.S., Balasundaram G., Chang Y.-T., Olivo M. (2014). Actively Targeted In Vivo Multiplex Detection of Intrinsic Cancer Biomarkers Using Biocompatible SERS Nanotags. Sci. Rep..

[99] Banaei N., Foley A., Houghton J.M., Sun Y., Kim B. (2017). Multiplex Detection of Pancreatic Cancer Biomarkers Using a SERS-Based Immunoassay. Nanotechnology.

[100] Lyu N., Rajendran V.K., Diefenbach R.J., Charles K., Clarke S.J., Engel A., Rizos H., Molloy M.P., Wang Y. (2020). Multiplex Detection of ctDNA Mutations in Plasma of Colorectal Cancer Patients by PCR/SERS Assay. Nanotheranostics.

[101] Saranya G., Joseph M.M., Karunakaran V., Nair J.B., Saritha V.N., Veena V.S., Sujathan K., Ajayaghosh A., Maiti K.K. (2018). Enzyme-Driven Switchable Fluorescence-SERS Diagnostic Nanococktail for the Multiplex Detection of Lung Cancer Biomarkers. ACS Appl. Mater. Interfaces.

[102] Plou J., García I., Charconnet M., Astobiza I., García-Astrain C., Matricardi C., Mihi A., Carracedo A., Liz-Marzán L.M. (2020). Multiplex SERS Detection of Metabolic Alterations in Tumor Extracellular Media. Adv. Funct. Mater..

[103] Lee S., Chon H., Yoon S.-Y., Kyu Lee E., Chang S.-I., Woo Lim D., Choo J. (2012). Fabrication of SERS-Fluorescence Dual Modal Nanoprobes and Application to Multiplex Cancer Cell Imaging. Nanoscale.

[104] Chen Z., Tabakman S.M., Goodwin A.P., Kattah M.G., Daranciang D., Wang X., Zhang G., Li X., Liu Z., Utz P.J. (2008). Protein Microarrays with Carbon Nanotubes as Multicolor Raman Labels. Nat. Biotechnol..

[105] Reza K.K., Dey S., Wuethrich A., Sina A.A.I., Korbie D., Wang Y., Trau M. (2018). Parallel Profiling of Cancer Cells and Proteins Using a Graphene Oxide Functionalized Ac-EHD SERS Immunoassay. Nanoscale.

[106] Chowdhury A.K.M.R.H., Tan B., Venkatakrishnan K. (2018). SERS-Active 3D Interconnected Nanocarbon Web toward Nonplasmonic in Vitro Sensing of HeLa Cells and Fibroblasts. ACS Appl. Mater. Interfaces.

[107] Banaei N., Moshfegh J., Mohseni-Kabir A., Marie Houghton J., Sun Y., Kim B. (2019). Machine Learning Algorithms Enhance the Specificity of Cancer Biomarker Detection Using SERS-Based Immunoassays in Microfluidic Chips. RSC Adv..

[108] Zhao J., Wang J., Liu Y., Han X.X., Xu B., Ozaki Y., Zhao B. (2022). Detection of Prostate Cancer Biomarkers via a SERS-Based Aptasensor. Biosens. Bioelectron..

[109] Zhang J., Dong Y., Zhu W., Xie D., Zhao Y., Yang D., Li M. (2019). Ultrasensitive Detection of Circulating Tumor DNA of Lung Cancer via an Enzymatically Amplified SERS-Based Frequency Shift Assay. ACS Appl. Mater. Interfaces.

[110] Wang J., Liang D., Jin Q., Feng J., Tang X. (2020). Bioorthogonal SERS Nanotags as a Precision Theranostic Platform for in Vivo SERS Imaging and Cancer Photothermal Therapy. Bioconjugate Chem..

[111] Li M., Ray Banerjee S., Zheng C., Pomper M.G., Barman I. (2016). Ultrahigh Affinity Raman Probe for Targeted Live Cell Imaging of Prostate Cancer. Chem. Sci..

[112] Murali V.P., Karunakaran V., Murali M., Lekshmi A., Kottarathil S., Deepika S., Saritha V.N., Ramya A.N., Raghu K.G., Sujathan K. (2023). A Clinically Feasible Diagnostic Spectro-Histology Built on SERS-Nanotags for Multiplex Detection and Grading of Breast Cancer Biomarkers. Biosens. Bioelectron..

[113] Mallia R.J., McVeigh P.Z., Fisher C.J., Veilleux I., Wilson B.C. (2015). Wide-Field Multiplexed Imaging of EGFR-Targeted Cancers Using Topical Application of NIR SERS Nanoprobes. Nanomedicine.

[114] Gao R., Cheng Z., deMello A.J., Choo J. (2016). Wash-Free Magnetic Immunoassay of the PSA Cancer Marker Using SERS and Droplet Microfluidics. Lab Chip.

[115] Ngo L., Pham L.Q.A., Tukova A., Hassanzadeh-Barforoushi A., Zhang W., Wang Y. (2023). Emerging Integrated SERS-Microfluidic Devices for Analysis of Cancer-Derived Small Extracellular Vesicles. Lab Chip.

[116] Știufiuc G.F., Toma V., Buse M., Mărginean R., Morar-Bolba G., Culic B., Tetean R., Leopold N., Pavel I., Lucaciu C.M. (2020). Solid Plasmonic Substrates for Breast Cancer Detection by Means of SERS Analysis of Blood Plasma. Nanomaterials.

[117] Romo-Herrera J.M., Juarez-Moreno K., Guerrini L., Kang Y., Feliu N., Parak W.J., Alvarez-Puebla R.A. (2021). Paper-Based Plasmonic Substrates as Surface-Enhanced Raman Scattering Spectroscopy Platforms for Cell Culture Applications. Mater. Today Bio.

[118] Guerrini L., Garcia-Rico E., O’Loghlen A., Giannini V., Alvarez-Puebla R.A. (2021). Surface-Enhanced Raman Scattering (SERS) Spectroscopy for Sensing and Characterization of Exosomes in Cancer Diagnosis. Cancers.

[119] Wang Z., Zong S., Wang Y., Li N., Li L., Lu J., Wang Z., Chen B., Cui Y. (2018). Screening and Multiple Detection of Cancer Exosomes Using an SERS-Based Method. Nanoscale.

[120] Xie Y., Su X., Wen Y., Zheng C., Li M. (2022). Artificial Intelligent Label-Free SERS Profiling of Serum Exosomes for Breast Cancer Diagnosis and Postoperative Assessment. Nano Lett..

[121] Moisoiu T., Dragomir M.P., Iancu S.D., Schallenberg S., Birolo G., Ferrero G., Burghelea D., Stefancu A., Cozan R.G., Licarete E. (2022). Combined miRNA and SERS Urine Liquid Biopsy for the Point-of-Care Diagnosis and Molecular Stratification of Bladder Cancer. Mol. Med..

[122] Liu X., Guo J., Li Y., Wang B., Yang S., Chen W., Wu X., Guo J., Ma X. (2021). SERS Substrate Fabrication for Biochemical Sensing: Towards Point-of-Care Diagnostics. J. Mater. Chem. B.

[123] Perumal J., Wang Y., Ebrahim Attia A.B., Dinish U.S., Olivo M. (2021). Towards a Point-of-Care SERS Sensor for Biomedical and Agri-Food Analysis Applications: A Review of Recent Advancements. Nanoscale.

[124] Hu R., Yong K.-T., Roy I., Ding H., He S., Prasad P.N. (2009). Metallic Nanostructures as Localized Plasmon Resonance Enhanced Scattering Probes for Multiplex Dark-Field Targeted Imaging of Cancer Cells. J. Phys. Chem. C.

[125] El-Safadi S., Tinneberg H.-R., von Georgi R., Münstedt K., Brück F. (2005). Does Dark Field Microscopy According to Enderlein Allow for Cancer Diagnosis? A Prospective Study. Forsch. Komplementarmedizin Klass. Naturheilkunde = Res. Complement. Nat. Class. Med..

[126] Gao P.F., Lei G., Huang C.Z. (2021). Dark-Field Microscopy: Recent Advances in Accurate Analysis and Emerging Applications. Anal. Chem..

[127] Zhang W., Du Q., Dou Z., Ning S., Wang Q., Dong Y., Yue Z., Ye W., Liu G. (2020). Ultrasensitive Detection of Lead (II) Ion by Dark-Field Spectroscopy and Glutathione Modified Gold Nanoparticles. Sens. Actuators B Chem..

[128] Guo Y., Liu F., Hu Y., Zheng X., Cao X., Zhu Y., Zhang X., Li D., Zhang Z., Chen S. (2020). Activated Plasmonic Nanoaggregates for Dark-Field in Situ Imaging for HER2 Protein Imaging on Cell Surfaces. Bioconjugate Chem..

[129] Liu M.L., Zou H.Y., Li C.M., Li R.S., Huang C.Z. (2017). Aptamer-Modified Selenium Nanoparticles for Dark-Field Microscopy Imaging of Nucleolin. Chem. Commun..

[130] Bhat A., Huan K., Cooks T., Boukari H., Lu Q. (2018). Probing Interactions between AuNPs/AgNPs and Giant Unilamellar Vesicles (GUVs) Using Hyperspectral Dark-Field Microscopy. Int. J. Mol. Sci..

[131] Cui Y., Wang X., Ren W., Liu J., Irudayaraj J. (2016). Optical Clearing Delivers Ultrasensitive Hyperspectral Dark-Field Imaging for Single-Cell Evaluation. ACS Nano.

[132] Crow M.J., Seekell K., Marinakos S., Ostrander J., Chilkoti A., Wax A.P. Hyperspectral Molecular Imaging of Multiple Receptors Using Immunolabeled Plasmonic Nanoparticles. Proceedings of the SPIE Conference, Plasmonics in Biology and Medicine VIII.

[133] Akagi T., Hanamura N., Ichiki T. (2015). Measurement of Individual Nanobioparticles on Microfluidic Chips by Laser Dark-Field Imaging. J. Photopolym. Sci. Technol..

[134] Yang Q., Cheng L., Hu L., Lou D., Zhang T., Li J., Zhu Q., Liu F. (2020). An Integrative Microfluidic Device for Isolation and Ultrasensitive Detection of Lung Cancer-Specific Exosomes from Patient Urine. Biosens. Bioelectron..

[135] Sikes J.C., Wonner K., Nicholson A., Cignoni P., Fritsch I., Tschulik K. (2022). Characterization of Nanoparticles in Diverse Mixtures Using Localized Surface Plasmon Resonance and Nanoparticle Tracking by Dark-Field Microscopy with Redox Magnetohydrodynamics Microfluidics. ACS Phys. Chem. Au.

[136] Sun D., Hu T.Y. (2018). A Low Cost Mobile Phone Dark-Field Microscope for Nanoparticle-Based Quantitative Studies. Biosens. Bioelectron..

[137] Nagy-Simon T., Tatar A.-S., Craciun A.-M., Vulpoi A., Jurj M.-A., Florea A., Tomuleasa C., Berindan-Neagoe I., Astilean S., Boca S. (2017). Antibody Conjugated, Raman Tagged Hollow Gold–Silver Nanospheres for Specific Targeting and Multimodal Dark-Field/SERS/Two Photon-FLIM Imaging of CD19(+) B Lymphoblasts. ACS Appl. Mater. Interfaces.

[138] Tan Z., Zhu C., Han L., Liao X., Wang C. (2022). SERS and Dark-Field Scattering Dual-Mode Detection of Intracellular Hydrogen Peroxide Using Biocompatible Au@COF Nanosensor. Sens. Actuators B Chem..

[139] Ip S., MacLaughlin C.M., Joseph M., Mullaithilaga N., Yang G., Wang C., Walker G.C. (2019). Dual-Mode Dark Field and Surface-Enhanced Raman Scattering Liposomes for Lymphoma and Leukemia Cell Imaging. Langmuir.

[140] Boyer D., Tamarat P., Maali A., Lounis B., Orrit M. (2002). Photothermal Imaging of Nanometer-Sized Metal Particles Among Scatterers. Science.

[141] He S., Song J., Qu J., Cheng Z. (2018). Crucial Breakthrough of Second Near-Infrared Biological Window Fluorophores: Design and Synthesis toward Multimodal Imaging and Theranostics. Chem. Soc. Rev..

[142] Kim M., Lee J.-H., Nam J.-M. (2019). Plasmonic Photothermal Nanoparticles for Biomedical Applications. Adv. Sci..

[143] Song J., Qu J., Swihart M.T., Prasad P.N. (2016). Near-IR Responsive Nanostructures for Nanobiophotonics: Emerging Impacts on Nanomedicine. Nanomed. Nanotechnol. Biol. Med..

[144] Thummerer G., Mayr G., Haltmeier M., Burgholzer P. (2020). Photoacoustic Reconstruction from Photothermal Measurements Including Prior Information. Photoacoustics.

[145] Skala M.C., Crow M.J., Wax A., Izatt J.A. (2008). Photothermal Optical Coherence Tomography of Epidermal Growth Factor Receptor in Live Cells Using Immunotargeted Gold Nanospheres. Nano Lett..

[146] Lapierre-Landry M., Gordon A.Y., Penn J.S., Skala M.C. (2017). In Vivo Photothermal Optical Coherence Tomography of Endogenous and Exogenous Contrast Agents in the Eye. Sci. Rep..

[147] Tucker-Schwartz J.M., Meyer T.A., Patil C.A., Duvall C.L., Skala M.C. (2012). In Vivo Photothermal Optical Coherence Tomography of Gold Nanorod Contrast Agents. Biomed. Opt. Express.

[148] Zeng Y., Zhang D., Wu M., Liu Y., Zhang X., Li L., Li Z., Han X., Wei X., Liu X. (2014). Lipid-AuNPs@PDA Nanohybrid for MRI/CT Imaging and Photothermal Therapy of Hepatocellular Carcinoma. ACS Appl. Mater. Interfaces.

[149] Zhou Z., Sun Y., Shen J., Wei J., Yu C., Kong B., Liu W., Yang H., Yang S., Wang W. (2014). Iron/Iron Oxide Core/Shell Nanoparticles for Magnetic Targeting MRI and near-Infrared Photothermal Therapy. Biomaterials.

[150] MacDonald T.D., Liu T.W., Zheng G. (2014). An MRI-Sensitive, Non-Photobleachable Porphysome Photothermal Agent. Angew. Chem..

[151] Alkilany A.M., Thompson L.B., Boulos S.P., Sisco P.N., Murphy C.J. (2012). Gold Nanorods: Their Potential for Photothermal Therapeutics and Drug Delivery, Tempered by the Complexity of Their Biological Interactions. Adv. Drug Deliv. Rev..

[152] Choi W.I., Sahu A., Kim Y.H., Tae G. (2012). Photothermal Cancer Therapy and Imaging Based on Gold Nanorods. Ann. Biomed. Eng..

[153] Yuan H., Khoury C.G., Wilson C.M., Grant G.A., Bennett A.J., Vo-Dinh T. (2012). In Vivo Particle Tracking and Photothermal Ablation Using Plasmon-Resonant Gold Nanostars. Nanomed. Nanotechnol. Biol. Med..

[154] Wang X., Li G., Ding Y., Sun S. (2014). Understanding the Photothermal Effect of Gold Nanostars and Nanorods for Biomedical Applications. RSC Adv..

[155] Pérez-Hernández M., del Pino P., Mitchell S.G., Moros M., Stepien G., Pelaz B., Parak W.J., Gálvez E.M., Pardo J., de la Fuente J.M. (2015). Dissecting the Molecular Mechanism of Apoptosis during Photothermal Therapy Using Gold Nanoprisms. ACS Nano.

[156] Moros M., Lewinska A., Merola F., Ferraro P., Wnuk M., Tino A., Tortiglione C. (2020). Gold Nanorods and Nanoprisms Mediate Different Photothermal Cell Death Mechanisms In Vitro and In Vivo. ACS Appl. Mater. Interfaces.

[157] Ambrosone A., del Pino P., Marchesano V., Parak W.J., de la Fuente J.M., Tortiglione C. (2014). Gold Nanoprisms for Photothermal Cell Ablation In Vivo. Nanomedicine.

[158] Chen M., Tang S., Guo Z., Wang X., Mo S., Huang X., Liu G., Zheng N. (2014). Core–Shell Pd@Au Nanoplates as Theranostic Agents for In-Vivo Photoacoustic Imaging, CT Imaging, and Photothermal Therapy. Adv. Mater..

[159] Wang J., Zhang Y., Liu L., Cui Z., Liu X., Wang L., Li Y., Li Q. (2019). Combined Chemo/Photothermal Therapy Based on Mesoporous Silica-Au Core-Shell Nanoparticles for Hepatocellular Carcinoma Treatment. Drug Dev. Ind. Pharm..

[160] Dai X., Li X., Du Y., Han M., Wang Z., Wang Y., Yan F., Liu Y. (2023). Gold Nanorod–Mesoporous Silica Core Shell Nanocomposites for NIR-II Photothermal Ablation and Dual PD-L1/VEGF Blockade Therapy in Hepatocellular Carcinoma. Chem. Eng. J..

[161] Li J., Hu Y., Yang J., Wei P., Sun W., Shen M., Zhang G., Shi X. (2015). Hyaluronic Acid-Modified Fe3O4@Au Core/Shell Nanostars for Multimodal Imaging and Photothermal Therapy of Tumors. Biomaterials.

[162] Hu R., Zheng M., Wu J., Li C., Shen D., Yang D., Li L., Ge M., Chang Z., Dong W. (2017). Core-Shell Magnetic Gold Nanoparticles for Magnetic Field-Enhanced Radio-Photothermal Therapy in Cervical Cancer. Nanomaterials.

[163] Ji M., Xu M., Zhang W., Yang Z., Huang L., Liu J., Zhang Y., Gu L., Yu Y., Hao W. (2016). Structurally Well-Defined Au@Cu2−xS Core–Shell Nanocrystals for Improved Cancer Treatment Based on Enhanced Photothermal Efficiency. Adv. Mater..

[164] Huang X., Jain P.K., El-Sayed I.H., El-Sayed M.A. (2008). Plasmonic Photothermal Therapy (PPTT) Using Gold Nanoparticles. Lasers Med. Sci..

[165] Austin L.A., Mackey M.A., Dreaden E.C., El-Sayed M.A. (2014). The Optical, Photothermal, and Facile Surface Chemical Properties of Gold and Silver Nanoparticles in Biodiagnostics, Therapy, and Drug Delivery. Arch. Toxicol..

[166] Singh A.K., Lu W., Senapati D., Khan S.A., Fan Z., Senapati T., Demeritte T., Beqa L., Ray P.C. (2011). Long-Range Nanoparticle Surface-Energy-Transfer Ruler for Monitoring Photothermal Therapy Response. Small.

[167] Fan Z., Shelton M., Singh A.K., Senapati D., Khan S.A., Ray P.C. (2012). Multifunctional Plasmonic Shell–Magnetic Core Nanoparticles for Targeted Diagnostics, Isolation, and Photothermal Destruction of Tumor Cells. ACS Nano.

[168] Shipunova V.O., Belova M.M., Kotelnikova P.A., Shilova O.N., Mirkasymov A.B., Danilova N.V., Komedchikova E.N., Popovtzer R., Deyev S.M., Nikitin M.P. (2022). Photothermal Therapy with HER2-Targeted Silver Nanoparticles Leading to Cancer Remission. Pharmaceutics.

[169] Zhou Z., Yan Y., Wang L., Zhang Q., Cheng Y. (2019). Melanin-like Nanoparticles Decorated with an Autophagy-Inducing Peptide for Efficient Targeted Photothermal Therapy. Biomaterials.

[170] Chen Z., Zhao P., Luo Z., Zheng M., Tian H., Gong P., Gao G., Pan H., Liu L., Ma A. (2016). Cancer Cell Membrane–Biomimetic Nanoparticles for Homologous-Targeting Dual-Modal Imaging and Photothermal Therapy. ACS Nano.

[171] Pu Y., Wu W., Zhou B., Xiang H., Yu J., Yin H., Zhang Y., Du D., Chen Y., Xu H. (2022). Starvation Therapy Enabled “Switch-on” NIR-II Photothermal Nanoagent for Synergistic in Situ Photothermal Immunotherapy. Nano Today.

[172] Liu Y., Tian J., Fu Y., Yang Y., Chen M., Zhang Q. (2021). Near-Infrared Light-Triggered Nanobomb for in Situ on-Demand Maximization of Photothermal/Photodynamic Efficacy for Cancer Therapy. Biomater. Sci..

[173] Jakobsohn K., Motiei M., Sinvani M., Popovtzer R. (2012). Towards Real-Time Detection of Tumor Margins Using Photothermal Imaging of Immune-Targeted Gold Nanoparticles. Int. J. Nanomed..

[174] Nedosekin D.A., Juratli M.A., Sarimollaoglu M., Moore C.L., Rusch N.J., Smeltzer M.S., Zharov V.P., Galanzha E.I. (2013). Photoacoustic and Photothermal Detection of Circulating Tumor Cells, Bacteria and Nanoparticles in Cerebrospinal Fluid In Vivo and Ex Vivo. J. Biophotonics.

[175] von Maltzahn G., Park J.-H., Agrawal A., Bandaru N.K., Das S.K., Sailor M.J., Bhatia S.N. (2009). Computationally Guided Photothermal Tumor Therapy Using Long-Circulating Gold Nanorod Antennas. Cancer Res..

[176] Lim C.-K., Shin J., Lee Y.-D., Kim J., Oh K.S., Yuk S.H., Jeong S.Y., Kwon I.C., Kim S. (2012). Phthalocyanine-Aggregated Polymeric Nanoparticles as Tumor-Homing Near-Infrared Absorbers for Photothermal Therapy of Cancer. Theranostics.

[177] Muthu M.S., Leong D.T., Mei L., Feng S.-S. (2014). Nanotheranostics ˗ Application and Further Development of Nanomedicine Strategies for Advanced Theranostics. Theranostics.

[178] Okuno T., Kato S., Hatakeyama Y., Okajima J., Maruyama S., Sakamoto M., Mori S., Kodama T. (2013). Photothermal Therapy of Tumors in Lymph Nodes Using Gold Nanorods and Near-Infrared Laser Light. J. Control. Release.

[179] Ong S.Y., Zhang C., Dong X., Yao S.Q. (2021). Recent Advances in Polymeric Nanoparticles for Enhanced Fluorescence and Photoacoustic Imaging. Angew. Chem. Int. Ed..

[180] Zhen X., Pu K., Jiang X. (2021). Photoacoustic Imaging and Photothermal Therapy of Semiconducting Polymer Nanoparticles: Signal Amplification and Second Near-Infrared Construction. Small.

[181] Mantri Y., Jokerst J.V. (2020). Engineering Plasmonic Nanoparticles for Enhanced Photoacoustic Imaging. ACS Nano.

[182] Manohar S., Ungureanu C., Van Leeuwen T.G. (2011). Gold Nanorods as Molecular Contrast Agents in Photoacoustic Imaging: The Promises and the Caveats. Contrast Media Mol. Imaging.

[183] Wang Y., Yang Y., Yang L., Lin Y., Tian Y., Ni Q., Wang S., Ju H., Guo J., Lu G. (2022). Gold Nanostar@Polyaniline Theranostic Agent with High Photothermal Conversion Efficiency for Photoacoustic Imaging-Guided Anticancer Phototherapy at a Low Dosage. ACS Appl. Mater. Interfaces.

[184] Manuel L.D.B., Vincely V.D., Bayer C.L., McPeak K.M. (2023). Monodisperse Sub-100 Nm Au Nanoshells for Low-Fluence Deep-Tissue Photoacoustic Imaging. Nano Lett..

[185] Chen Y., Xu C., Cheng Y., Cheng Q. (2021). Photostability Enhancement of Silica-Coated Gold Nanostars for Photoacoustic Imaging Guided Photothermal Therapy. Photoacoustics.

[186] Chen Y.-S., Frey W., Kim S., Kruizinga P., Homan K., Emelianov S. (2011). Silica-Coated Gold Nanorods as Photoacoustic Signal Nanoamplifiers. Nano Lett..

[187] Chen Y.-S., Frey W., Kim S., Homan K., Kruizinga P., Sokolov K., Emelianov S. (2010). Enhanced Thermal Stability of Silica-Coated Gold Nanorods for Photoacoustic Imaging and Image-Guided Therapy. Opt. Express.

[188] Galanzha E.I., Shashkov E.V., Kelly T., Kim J.-W., Yang L., Zharov V.P. (2009). In Vivo Magnetic Enrichment and Multiplex Photoacoustic Detection of Circulating Tumour Cells. Nat. Nanotechol..

[189] Santiesteban D.Y., Hallam K.A., Yarmoska S.K., Emelianov S.Y. (2019). Color-Coded Perfluorocarbon Nanodroplets for Multiplexed Ultrasound and Photoacoustic Imaging. Nano Res..

[190] Luke G.P., Myers J.N., Emelianov S.Y., Sokolov K.V. (2014). Sentinel Lymph Node Biopsy Revisited: Ultrasound-Guided Photoacoustic Detection of Micrometastases Using Molecularly Targeted Plasmonic Nanosensors. Cancer Res..

[191] Liu X., Duan Y., Liu B. (2021). Nanoparticles as Contrast Agents for Photoacoustic Brain Imaging. Aggregate.

[192] Li H., Shi S., Wu M., Shen W., Ren J., Mei Z., Ran H., Wang Z., Tian Y., Gao J. (2021). iRGD Peptide-Mediated Liposomal Nanoparticles with Photoacoustic/Ultrasound Dual-Modality Imaging for Precision Theranostics Against Hepatocellular Carcinoma. Int. J. Nanomed..

[193] Chen J., Zeng S., Xue Q., Hong Y., Liu L., Song L., Fang C., Zhang H., Wang B., Sedgwick A.C. (2022). Photoacoustic Image-Guided Biomimetic Nanoparticles Targeting Rheumatoid Arthritis. Proc. Natl. Acad. Sci. USA.

[194] Zhong H., Jiang D., Lan H., Duan T., Gao F., Gao F. (2020). Low-Cost Multi-Wavelength Photoacoustic Imaging Based on Portable Continuous-Wave Laser Diode Module. IEEE Trans. Biomed. Circuits Syst..

[195] Wu X., Sanders J.L., Dundar M.M., Oralkan Ö. (2023). Deep-Learning-Based High-Intensity Focused Ultrasound Lesion Segmentation in Multi-Wavelength Photoacoustic Imaging. Bioengineering.

[196] Zhang Y.-J., Guo L., Chen S., Yu Y.-L., Wang J.-H. (2020). A Portable Photoacoustic Device for Facile and Sensitive Detection of Serum Alkaline Phosphatase Activity. Anal. Chim. Acta.

[197] Wang H., Liu S., Wang T., Zhang C., Feng T., Tian C. (2019). Three-Dimensional Interventional Photoacoustic Imaging for Biopsy Needle Guidance with a Linear Array Transducer. J. Biophotonics.

[198] Xia W., West S.J., Nikitichev D.I., Ourselin S., Beard P.C., Desjardins A.E. Interventional Multispectral Photoacoustic Imaging with a Clinical Linear Array Ultrasound Probe for Guiding Nerve Blocks. Proceedings of the SPIE Conference, Photons Plus Ultrasound: Imaging and Sensing 2016.

[199] Liu R., Jing L., Peng D., Li Y., Tian J., Dai Z. (2015). Manganese (II) Chelate Functionalized Copper Sulfide Nanoparticles for Efficient Magnetic Resonance/Photoacoustic Dual-Modal Imaging Guided Photothermal Therapy. Theranostics.

[200] Zhang Q., Iwakuma N., Sharma P., Moudgil B.M., Wu C., McNeill J., Jiang H., Grobmyer S.R. (2009). Gold Nanoparticles as a Contrast Agent for In Vivo Tumor Imaging with Photoacoustic Tomography. Nanotechnology.

[201] Chen J., Nguyen V.P., Jaiswal S., Kang X., Lee M., Paulus Y.M., Wang T.D. (2021). Thin Layer-Protected Gold Nanoparticles for Targeted Multimodal Imaging with Photoacoustic and CT. Pharmaceuticals.

[202] Jo J., Lee C.H., Kopelman R., Wang X. (2017). In Vivo Quantitative Imaging of Tumor pH by Nanosonophore Assisted Multispectral Photoacoustic Imaging. Nat. Commun..

[203] Haisch C. (2016). Raman-Based Microarray Readout: A Review. Anal. Bioanal. Chem..

[204] Nguyen N.T.T., Yaraki M.T., Wang Y. (2023). Enabling Spectral Barcoding of SERS Nanotags Using Gold Nanostars. Mol. Syst. Des. Eng..

[205] Xiao R., Lu L., Rong Z., Wang C., Peng Y., Wang F., Wang J., Sun M., Dong J., Wang D. (2020). Portable and Multiplexed Lateral Flow Immunoassay Reader Based on SERS for Highly Sensitive Point-of-Care Testing. Biosens. Bioelectron..

[206] Li X., Yang T., Li C.S., Song Y., Lou H., Guan D., Jin L. (2018). Surface Enhanced Raman Spectroscopy (SERS) for the Multiplex Detection of Braf, Kras, and Pik3ca Mutations in Plasma of Colorectal Cancer Patients. Theranostics.

[207] Nyayapathi N., Xia J. (2019). Photoacoustic Imaging of Breast Cancer: A Mini Review of System Design and Image Features. J. Biomed. Opt..

[208] García-Hernández L.A., Martínez-Martínez E., Pazos-Solís D., Aguado-Preciado J., Dutt A., Chávez-Ramírez A.U., Korgel B., Sharma A., Oza G. (2023). Optical Detection of Cancer Cells Using Lab-on-a-Chip. Biosensors.

[209] Marquez S., Morales-Narváez E. (2019). Nanoplasmonics in Paper-Based Analytical Devices. Front. Bioeng. Biotechnol..

[210] Ge S., Chen G., Deng J., Gu Y., Mao Y., Zhou X., Li G. (2023). Multiplex Signal Amplification Strategy-Based Early-Stage Diagnosis of Parkinson’s Disease on a SERS-Enabled LoC System. Anal. Chim. Acta.

[211] Fan Z., Geng Z., Fang W., Lv X., Su Y., Wang S., Chen H. (2020). Smartphone Biosensor System with Multi-Testing Unit Based on Localized Surface Plasmon Resonance Integrated with Microfluidics Chip. Sensors.

[212] Pinheiro T., Marques A.C., Carvalho P., Martins R., Fortunato E. (2021). Paper Microfluidics and Tailored Gold Nanoparticles for Nonenzymatic, Colorimetric Multiplex Biomarker Detection. ACS Appl. Mater. Interfaces.

[213] He J.-L., Wang D.-S., Fan S.-K. (2016). Opto-Microfluidic Immunosensors: From Colorimetric to Plasmonic. Micromachines.

[214] Cong S., Yuan Y., Chen Z., Hou J., Yang M., Su Y., Zhang Y., Li L., Li Q., Geng F. (2015). Noble Metal-Comparable SERS Enhancement from Semiconducting Metal Oxides by Making Oxygen Vacancies. Nat. Commun..

[215] Zhang B.Y., Yin P., Hu Y., Szydzik C., Khan M.W., Xu K., Thurgood P., Mahmood N., Dekiwadia C., Afrin S. (2022). Highly Accurate and Label-Free Discrimination of Single Cancer Cell Using a Plasmonic Oxide-Based Nanoprobe. Biosens. Bioelectron..

